# Advancing automated cell type annotation with large language models and single-cell isoform sequencing

**DOI:** 10.1016/j.csbj.2025.11.008

**Published:** 2025-11-06

**Authors:** Hettiarachchige Wijewardena, Saloni Bhatia, Namrata Bhattacharya, Debarka Sengupta, Siyuan Wu, Ulf Schmitz

**Affiliations:** a Computational Biomedicine Lab, College of Science and Engineering, James Cook University, Townsville, QLD, Australia; b Australian Prostate Cancer Research Centre-Queensland, Faculty of Health, School of Biomedical Sciences, Centre for Genomics and Personalised Health, Queensland University of Technology, Brisbane, QLD 4000, Australia; c Department of Computer Science and Engineering, Indraprastha Institute of Information Technology-Delhi (IIIT-Delhi), Okhla, Phase III, New Delhi 110020, India; d Translational Research Institute, Princess Alexandra Hospital, Woolloongabba, QLD 4102, Australia; e Department of Computational Biology, Indraprastha Institute of Information Technology-Delhi (IIIT-Delhi), Okhla, Phase III, New Delhi 110020, India; f Centre for Artificial Intelligence, Indraprastha Institute of Information Technology-Delhi (IIIT-Delhi), Okhla, Phase III, New Delhi 110020, India; g School of Mathematics, Monash University, Melbourne, Victoria 3800, Australia; h Centre for Tropical Bioinformatics and Molecular Biology, Australian Institute of Tropical Health and Medicine, James Cook University, Cairns, Australia; i Centenary Institute, The University of Sydney, Camperdown, Australia

**Keywords:** Single-cell RNA sequencing, Automatic cell type annotation, Machine learning, Transcript isoforms, Alternative splicing, Large language models, Natural language processing

## Abstract

Accurate cell type identification is critical for interpreting single-cell transcriptomic data and understanding complex biological systems. In this review, we discuss how natural language processing and large language models can enhance the accuracy and scalability of cell type annotation. We also highlight how emerging single-cell long-read sequencing technologies enable isoform-level transcriptomic profiling, offering higher resolution than conventional gene expression-based methods and providing opportunities to redefine cell types. By integrating the insights of key technical and algorithmic advances across sequencing and computational approaches, we provide a unified overview of recent developments that are reshaping automated cell type annotation and improving the precision of biological interpretation.

## Graphical Abstract

**Figure:**
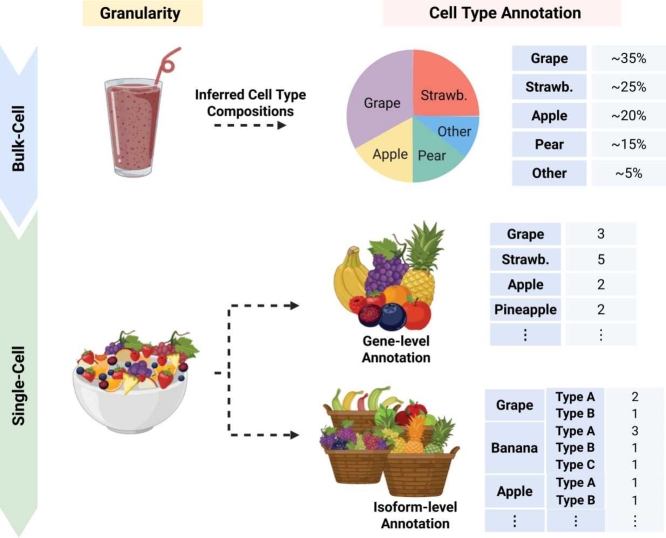

## 1 Introduction

Cells are the fundamental units of life. The ensemble of expressed genes and proteins in a cell defines its identity and function, or phenotype, at any specific point in time. It is therefore no surprise that anomalies in gene expression are an observable manifestation of many diseases. Likewise, while orchestrated transcript isoforms expression enables the regulation of critical biological processes, abnormal isoform expression has been linked to illnesses such as cancer [1].

Consequently, knowing the canonical transcriptome profile of cell types can facilitate the identification of early signs of diseases and the discovery of therapeutic targets. Conversely, precise identification of cell types is essential for cellular engineering, including somatic cell reprogramming [2], guided differentiation of pluripotent stem cells [3], and direct conversions among differentiated cell lineages [4], such as therapeutic T-cell engineering [5].

Single-cell sequencing enables high-throughput transcriptomic profiling of thousands of individual cells from a tissue or organism in parallel. This is achieved using droplet-, plate-, or microwell-based approaches combined with second- or third-generation sequencing technologies, allowing transcriptomic analysis at the gene, isoform, or even spatial resolution [6], [7].

### 1.1 Cell type annotation

Cell type annotation facilitates the assignment of an identity to cellular transcriptomic profiles, which can be grouped to allow for inter-cell-type or cross-sample comparison. Conventional cell type annotation in single-cell transcriptomic data relies on expert manual labelling of cell clusters using known marker genes and biological insight [8]. It typically involves two steps: clustering cells based on transcriptomic similarity, followed by assigning cell type labels by comparing marker gene expression to references [9]. Annotators often need to consult literature and mine existing data to identify context-specific markers, especially when canonical markers are insufficient or ambiguous [8], [9]. Although effective, this process is slow, labour-intensive, and requires both computational and domain expertise, and because it is often not based on standardized cell label ontologies, it is difficult to reproduce [8], [10].

As a consequence, automatic cell type annotation systems have advanced in recent years. In this context, machine learning (ML) can significantly improve the understanding of the cellular composition of tissues from single-cell RNA sequencing data [11]. This entails analysing the data to understand the structure and composition of tissue at the cellular level, capturing the interrelationships of cell markers, and developing models of cell populations. ML enables the identification of diverse cell types within complex tissues across different species, provided that suitable training datasets are available, thereby facilitating the reconstruction of cellular networks.

### 1.2 Natural language processing

ML has witnessed the remarkable impact of transformers, which were initially developed for natural language processing (NLP) tasks. NLP encompasses computational approaches that enable the representation and interpretation of human language, converting unstructured text into analysable forms that ML algorithms can process more effectively. These have now enabled NLP-based cell characterisation methods that significantly expand the scope of automated cell type annotation, allowing for more nuanced identification of cell subpopulations and rare cell types [12]. Building on these methods, the development of large language models (LLMs) has further enhanced this capability. LLMs are accessed via provider-specific interfaces or open-source implementations and serve as the core reasoning component of AI systems, supported by mechanisms such as memory and tool use [13], [14]. By utilizing extensive training datasets, LLMs can assist in the identification of complex cell types and their characteristics [15]. However, challenges remain, particularly with reproducibility, and the fact that LLMs are not specifically designed for cell type annotation [16].

### 1.3 Third-generation sequencing

A recent uptake of single-cell long-read sequencing studies have allowed studying single-cell transcriptomes at finer granularity. Long-read sequencing or isoform sequencing (a.k.a. third-generation sequencing, TGS) technologies facilitate the investigation of full-length isoforms and transcriptomic complexity patterns [17], [18]. Pacific Biosciences (PacBio) and Oxford Nanopore Technologies (ONT) are two prominent platforms for TGS that enable sequencing of fragments exceeding 10 kb in length.

However, cell type annotation methods have not yet fully leveraged the enhanced transcriptomic resolution to improve classification performance and enable the identification of novel or rare cell types. This review examines recent developments in automated cell type classification, single-cell RNA sequencing technologies, and the potential for LLMs and long-read sequencing to further advance the field.

**Fig. 1:**
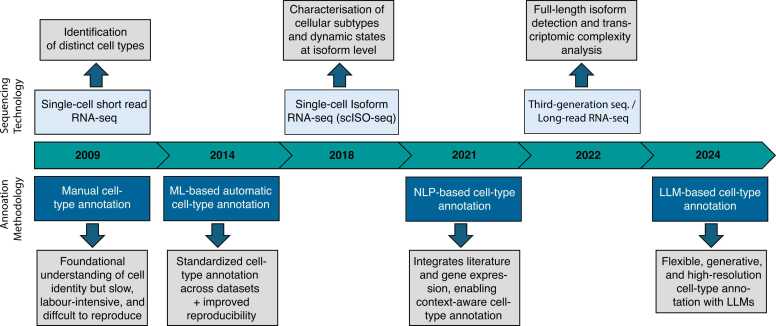
Timeline of key milestones in the evolution of single-cell RNA-seq–based cell type annotation. The timeline highlights major technological and algorithmic advances shaping the field of cell type annotation. Single-cell RNA sequencing (scRNA-seq) was first introduced in 2009 [116], enabling the detection of cellular heterogeneity previously masked in bulk RNA-seq, followed by manual annotation approaches based on marker genes. ML-based automatic annotation tools were subsequently developed [117], enhancing reproducibility and scalability across datasets. The introduction of single-cell isoform sequencing [82] allowed the characterization of cellular subtypes and dynamic states at the isoform level. Later, natural language processing (NLP)-based methods such as *scETM*[56] and *CellMeSH*[57] integrated literature and gene expression information for context-aware annotation. Advances in third-generation long-read sequencing technologies (PacBio, Nanopore) [118] further enabled full-length isoform detection. Most recently, the emergence of large language model (LLM)-based frameworks [59], [63] has facilitated flexible, generative, and high-resolution approaches to cell type annotation.

See major milestone in the evolution of cell type annotation in Fig. 1.

## 2 Machine-learning models for automated cell type classification

Automation of cell type identification eliminates the need for manual annotation, making it accessible to those unfamiliar with cell markers while also saving resources when working with large datasets [19], [20]. As a result, there has been a surge in the development of computational tools over the past decade, specifically designed to automate cell type identification from single-cell RNA sequencing data (Supplementary Table 1).

These tools can be broadly classified into two main categories: (1) Reference-based methods utilise existing datasets or models to classify new cells. These methods include supervised approaches that require explicit model training on labelled datasets, unsupervised approaches that compare new cells to reference profiles based on patterns learned from unlabelled datasets, and pretrained classifiers adapted from NLP or LLMs. (2) Marker-based methods rely on predefined marker genes or cell type signatures from biological databases and ontologies, assigning cell identities based on marker expression patterns without large annotated references.

Within the reference-based category, most tools integrate ML techniques, ranging from traditional classifiers such as neural networks (NN), random forests (RF), and support vector machines (SVM), to ensemble and ML-based hybrid approaches. A recent trend involves composite models combining multiple ML algorithms to enhance performance, robustness, and interpretability (e.g., Moana [21], *scReClassify* [22], or *ImmClassifier* [23]). While many non-ML methods still rely on statistical or rule-based frameworks, some hybrid tools like Digital Cell Sorter [24] incorporate ML within marker-based approaches.

To assess the effectiveness of these diverse approaches, multiple benchmark studies have compared the performance of cell type annotation tools and ML models (Table 1). Despite the diversity of strategies, marker-based methods do not consistently outperform others, underscoring the importance of robust marker gene selection [10]. Abdelaal et al., Xie et al., and Hung et al. benchmarked both reference-based and marker-based methods, whereas Zhao et al. evaluated only reference-based approaches. In contrast, Huang and Zhang et al. and Tortelote et al. focused on benchmarking general-purpose ML models for cell type annotation.

**Table 1:** Benchmark studies comparing the performance of cell type annotation tools and ML models.

**Study**	**Notes**	**Reference**
Abdelaal et al.	Evaluated the efficacy of 22 different approaches for automated cell identification with 27 single-cell RNA sequencing datasets.	[10]
Xie et al.	Assessed 32 existing automated cell type identification techniques for scRNA-seq, examining their efficacy across various datasets.	[19]
Huang and Zhang	Performed an extensive assessment of 10 ML models for the automatic assignment of cell phenotypes utilizing 20 publically accessible scRNA-seq datasets.	[25]
Hung et al.	Evaluated eleven cell type annotation methodologies, accessible as R packages, examining their efficacy across a diverse array of public scRNA-seq datasets and simulated data.	[26]
Zhao et al.	Assessed nine classification algorithms tailored for scRNA-seq datasets utilising three distinct sources which contains highly credible cell type labels.	[27]
Tortelote	In a preprint article, Tortelote assessed the efficacy of eight ML models for cell annotation in scRNA-seq versus single-nucleus RNA sequencing (snRNA-seq) datasets.	[28]

Benchmark studies show that cell type annotation tools vary widely in performance depending on dataset complexity, tissue type, and cell-type similarity. Tools such as *scPred* [29], *SingleCellNet* [30], *ACTINN* [31], *CaSTLe* [32], *scmapcell* [33], *SingleR* [34], and *SCINA* [35] achieve near-perfect accuracy with well-structured datasets but struggle with heterogeneous or complex datasets [10], [27], while *Cell BLAST* [36], *CHETAH* [37], and *scVI* [38] often underperform in these settings. General-purpose ML models such as SVM and RF perform well with low-complexity datasets but decline in performance when faced with large or overlapping cell populations [10], [25], [39]. Across all benchmarks, no single tool consistently outperforms others, and runtime differences reflect trade-offs between computational cost and algorithmic complexity [27], [33], [34], [40]

Notably, most automated cell type classification tools are optimised for short-read sequencing data. Future efforts should focus more on adapting models for isoform-resolved annotation using long-read RNA sequencing data.

Detailed descriptions of the datasets used in the cell type annotation benchmark studies discussed here are provided in Supplementary Table 2.

Even though traditional ML–based approaches play a crucial role in automating single-cell type annotation, they often face dataset-specific challenges that limit their generalisability and interpretability, which was also the observation in recent cell type annotation benchmark studies [15]. ML-based models typically rely on reference datasets, making their performance highly dependent on the quality and representativeness of the training data. Consequently, ML-based methods tend to perform well within specific tissues or experimental conditions but exhibit reduced accuracy when applied to novel or heterogeneous datasets [14], [15]. Moreover, the high dimensionality and nonlinear relationships inherent in mRNA expression data pose additional challenges. Most ML pipelines rely on highly variable gene (HVG) selection and dimensionality reduction techniques such as PCA, which risk discarding biologically relevant information, especially for rare cell types, and introduce artificial biases due to parameter sensitivity [29], [31], [41], [42], [43], [44]. The selected HVGs are often dataset-specific and may not capture co-expression or biological interactions of genes which are critical for accurate annotation [45]. These limitations collectively restrict their ability to generalise beyond the training datasets and discover new cell types. In contrast, LLMs have been introduced to address these issues by reframing transcriptomic data within a natural language context, allowing models to capture complex gene–gene relationships, contextual dependencies, and nonlinear patterns [46]. By leveraging pretraining on large, diverse biological corpora, LLMs can overcome the narrow scope and limited scalability of ML-based annotation methods [43], [47], [48].

## 3 Large language model-based cell type annotation

LLMs, particularly transformer-based foundation models, offer a unified framework capable of learning from massive, heterogeneous datasets, enabling broader applicability across tissues and conditions [47].

Foundation models, is a category of large scale pre-trained models that are characterised by their scalability and adaptability. Trained on vast datasets, they capture complex biological patterns and can be reused across diverse tasks with minimal modification. However, this power comes with high computational demands, often requiring GPU or TPU clusters and involving billions of parameters [49].

LLMs evolved from traditional ML methods through breakthroughs in deep learning, specifically the transformer architecture, which uses self-attention, an attention mechanism that relates different positions of a single sequence to compute a context-aware representation of the sequence, to capture long-range dependencies in sequential data [50], [51], [52]. Even though transformers were originally developed for NLP, they have now underlined the foundation models in biology for LLMs, where genes are treated as tokens in a sequence, much like words in a sentence [49], [53].

In the context of single-cell biology, LLMs facilitate automated cell type annotation by embedding gene expression data into meaningful representations. Drawing parallels to language, where texts comprise words, cells are defined by genes, though this remains an analogy rather than a direct equivalence - it simply explains the complex relationship [47]. This enables the possibility of classification of cell subtypes and rare populations, even across batches or modalities [49], [54]. By modelling genes as textual tokens, LLMs can continuously improve with new data, easily incorporate additional features and metadata, and uncover subtle functional relationships and developmental trajectories [47].

LLMs are trained in two stages: pretraining and fine-tuning. In pretraining, the model learns general patterns from large-scale unlabelled single-cell datasets using self-supervised objectives. Cell representations are often learned via special tokens or via a cell embedding matrix derived from the model’s output. During fine-tuning, these pretrained embeddings are adapted to specific downstream tasks including cell type annotation, perturbation prediction, or integration. This two-phase training enables LLMs to generalise well while retaining task-specific precision [49], [53].

Pretrained on comprehensive atlas-level datasets such as PanglaoDB (panglaodb.se) [55] and the Human Cell Atlas (www.humancellatlas.org), models like *scBERT* [43] integrate database-driven annotation tools with generative artificial intelligence (AI). These models combine multiple annotation techniques and apply self-iterative optimisation, selecting the best strategies for each case and improving accuracy and interpretability [14], [56]. As a result, LLMs facilitate precise annotation of rare cell types and reveal complex differentiation trajectories previously difficult to resolve [54].

Recent advancements in NLP- and LLM-based cell type annotation have led to the development of a growing number of software tools. Among them, *MarkerGeneBERT*, a direct application of NLP, systematically extracts cell type and marker gene information from scientific literature to enhance the accuracy and efficiency of scRNA-seq based cell type annotation [12]. *CellMeSH*, on the other hand, automatically constructs a comprehensive gene–cell-type association database from indexed biomedical literature and uses a probabilistic querying approach to predict cell types from scRNA-seq clusters, enabling scalable and literature-informed annotation [57]. Although tools such as *SCellBOW* [58] and *scETM* [56] do not directly annotate cell types, they provide a crucial supportive role. *SCellBOW*, an unsupervised transfer learning method, treats genes as words and cells as documents to enable clustering, phenotypic analysis, and the detection of malignant subpopulations [58]. *scETM* on the other hand employs embedded topic modeling and NNs to evaluate scRNA-seq data, providing interpretable gene embeddings and zero-shot transfer learning across many tissues and species [56]. Table 2 provides a comparative summary of LLM-based tools developed for cell type annotation. Supplementary Table 3 presents detailed information on each LLM based cell type annotation tool, including input modalities, training data sources and sizes, model interpretability, computational cost, and biological validation strategies. Supplementary Table 4 lists the datasets used to evaluate annotation accuracy and specifies the corresponding cell types analysed in each study.

**Table 2:** LLM-based cell type annotation tools.

Software	**Description**	**Reference**
*LICT*	A software package that employs a multi-model fusion and "talk-to-machine" technique to enhance annotation reliability, particularly in datasets characterised by low cellular heterogeneity.	[15]
*GPTCelltype*	An R package integrating *ChatGPT* powered by *GPT−4*, which enables LLMs to execute cell-type annotations independently, without extensive domain knowledge or reference datasets.	[16]
*CellAgent*	A multi-agent framework leveraging LLMs to automate single-cell RNA sequencing data analysis and cell type annotation, delivering high-quality results without human intervention.	[14]
*CASSIA*	A multi-agent LLM-based tool that enhances annotation accuracy while improving interpretability by providing reasoning and quality scores for each prediction.	[59]
*Celler*	*Celler* is a transformer-based annotation tool that applies LLM concepts to improve the identification of rare and disease-relevant cell types. Using innovations like GInf Loss and Hard Data Mining, it effectively handles long-tailed distributions in single-cell data.	[46]
*CellReasoner*	A lightweight, open-source LLM for single-cell type annotation that maps gene expression profiles to cell types with strong generalisation and interpretable, marker-level reasoning	[60]
*CellTypeAgent*	LLM-based annotation tool that uses marker genes and integrates GPT models with curated databases to improve accuracy and reduce hallucinations, enabling efficient and reliable cell type identification.	[9]
*ReCellTy*	A retrieval-augmented LLM framework for single-cell annotation that leverages a structured knowledge graph built from a refined CellMarker2.0 database.	[61]
*scBERT*	A transformer-based model inspired by LLMs such as *BERT*, repurposed for single-cell RNA-seq data. It leverages pretraining on large-scale unlabelled gene expression data followed by fine-tuning to accurately annotate cell types.	[43]
*scExtract*	An automated framework for single-cell RNA-seq that uses LLMs to extract insights from research articles, guiding data processing, integration, and annotation for large-scale meta-analysis with minimal manual effort.	[62]
*scInterpreter*	Harnesses the broad biological knowledge and reasoning capabilities of LLMs to interpret and classify cell types from gene expression data, demonstrating the value of integrating general-domain knowledge into single-cell analysis.	[63]
*scGPT*	Transformer-based foundation model that learns gene and cell embeddings from over 33 million cells, enabling cell type annotation, data integration, and perturbation prediction, offering high accuracy and interpretability.	[47], [64]

Besides dedicated cell type annotation tools, several specialized single-cell foundation models also enable cell type annotation, even though it is not their primary purpose. *CellPLM* is a pre-trained single-cell language model capturing cell–cell relationships and spatial transcriptomic patterns, supporting downstream tasks including clustering, perturbation prediction, and cell type annotation [65]. *Geneformer* models gene network dynamics from large-scale single-cell transcriptomic data and identifies candidate therapeutic targets, while also facilitating cell type annotation [66]. *GenePT* leverages LLM-derived gene embeddings to represent gene–cell relationships, making it applicable for cell type identification [67]. *Cell2Text* is a multimodal generative model that produces interpretable natural-language descriptions from single-cell RNA-seq data, with the additional capability of annotating cell types [68].

### 3.1 Benchmarks of LLM-based cell type annotation algorithms

SOAR is a large-scale benchmarking study that has evaluated the performance of eight instruction-tuned LLMs (*DeepSeek-LLM-67B, Qwen2–72B, Llama-3–70B, Mixtral-8 ×7B, Mixtral-8 ×22B, Cell2Sentence, GPT-4o mini, GPT-4o*) across eleven datasets for cell type annotation in single-cell genomics. Their findings highlighted that LLMs exhibit strong interpretive capabilities in scRNA-seq data without extensive fine-tuning, while also showing promising potential for cross-modality analysis in multi-omics contexts [69]. Additionally, AnnDictionary has been utilised to assess commercially available LLMs for cell type annotation using the Tabula Sapiens datasets [13], [70]. This study demonstrated that LLM annotation of most primary cell types achieves an accuracy exceeding 80–90 %. A leaderboard for LLM cell type annotation, based on evaluations using Tabula Sapiens data, is available at https://singlecellgpt.com/celltype-annotation-leaderboard [13].

### 3.2 Advantages and challenges of LLM-based models for cell type annotation

Transformer-based LLMs show strong generalisation to unseen data and support user-guided annotation via chatbot interfaces [53]. Pretrained on large-scale datasets, they leverage attention mechanisms to encode prior biological knowledge, enabling batch-insensitive annotations across diverse tissues, species, and technologies [49], [53]. LLMs facilitate reference-query data integration without needing explicit batch labels and can handle intra- and inter-dataset predictions [49]. They offer improved annotation consistency compared to manual methods and enable end-to-end analysis without additional fine-tuning, enhancing scalability and automation in single-cell genomics [48].

LLM-based models offer several advantages over traditional ML-based methods by enhancing scalability, interpretability, and accessibility in cell type annotation. For instance, *GPT-4* demonstrates both cost-efficiency and seamless integration with existing single-cell analysis frameworks such as Seurat, removing the need for separate pipelines or curated reference datasets [16]. Its broad pretraining across extensive datasets enables robust generalisation across tissues and species, while its interactive chatbot nature allows for dynamic, user-guided annotation refinement [9], [16]. Beyond single-agent models, multi-agent frameworks like *CASSIA* extend these benefits by providing automated, accurate, and interpretable annotations, along with annotation-specific quality scores that flag uncertain predictions for manual review or model-based correction. Benchmarking studies have shown that such frameworks outperform traditional ML and semi-automated methods, particularly in complex datasets from cancer, immunology, and rare species [59]. Moreover, the capacity of LLMs to capture long-range dependencies and contextual associations between genes through transformer architectures enables improved modelling compared to ML methods that rely on linear dimensionality reduction and dataset-specific feature selection approaches [43]. Collectively, these advances illustrate the transformative potential of LLM-based annotation systems to deliver higher accuracy, broader applicability, and reduced reliance on expert intervention compared to conventional ML-based tools.

Despite their strengths, LLMs face several limitations. General-purpose LLMs such as GPT remain limited in biological applications [59]. Their lack of domain-specific pretraining often results in biologically irrelevant outputs, limited interpretability, and suboptimal performance in tasks like cell type annotation [10], [71]. High-quality and context-diverse pretraining datasets, spanning different cell types, disease states, tissues, genders, and species, are essential for improving the generalisability and biological relevance of single-cell LLMs [72], [73]. Incorporating quality verification strategies, such as online learning frameworks, can further refine dataset selection and ensure robust model performance across downstream tasks [74]. These models are also difficult to deploy due to their scale and reliance on proprietary application programming interfaces (APIs) [60]. For example, while *GPT-4* performs well on peripheral blood mononuclear cell (PBMC) and gastric cancer datasets, it struggles with more complex data like human embryonic cells. Similarly, *ERNIE 4.0* shows high accuracy in low-heterogeneity datasets, such as stromal cells, but lacks generalisability [15]. These shortcomings have prompted the development of domain-adapted solutions. Agent-based frameworks, such as *CellAgent* [14], integrate LLMs with bioinformatics tools in structured workflows, enabling context-aware, interpretable analysis of scRNA-seq data [75]. Fine-tuning LLMs on curated marker gene databases further improves their biological relevance and task-specific accuracy [16].

Integrating language-based models with omics data, structurally distinct from natural language, is inherently complex [53]. Evaluation is further complicated by non-deterministic outputs and the evolving nature of proprietary models like *GPT-4*. A lack of transparency in training data, biases, and hallucinations, plausible yet factually incorrect outputs that can skew scientific conclusions and undermine accuracy, pose significant concerns [48]. LLMs also struggle with rare or novel cell types, low-heterogeneity data, skewed cell type distributions, and rigid input formats. Annotation quality is often influenced by training data bias, and outputs typically require expert validation [16], [48]. High computational costs, environmental impact, and the need for extensive tuning limit reproducibility and accessibility. Overreliance on LLMs without rigorous human oversight increases the risk of misinterpretation in downstream analyses. [48], [53].

While ethical issues are relatively limited at this stage, as most LLM-based cell type annotation tools operate as standalone systems, reproducibility remains an ongoing challenge [15]. However, many recent tools have begun integrating mechanisms to improve transparency and consistency of results [14], [46], [60]. The opacity often associated with proprietary LLMs has been partially mitigated by developers openly disclosing training data sources, model architectures, and fine-tuning strategies, as summarized in Supplementary Table 3. For instance, *CASSIA* enhances interpretability by providing validator cross-checks, quantitative quality scores, and HTML reports documenting each decision [59], *CellReasoner* employs reasoning-augmented annotation through chain-of-thought (CoT) supervision that combines AI- and human-curated reasoning paths [60], and *ReCellTy* offers intermediate reasoning steps and transparent annotation tracing linked to selected features [61]. Moreover, most tools explicitly report their training datasets, improving data provenance and traceability [43], [46]. Nevertheless, challenges persist when these models rely on general-purpose LLMs trained on undisclosed or heterogeneous biomedical corpora, where complete data transparency and ethical assurance remain difficult to guarantee [14], [16].

## 4 Cell type annotation with isoform-resolved transcriptomics

**Fig. 2:**
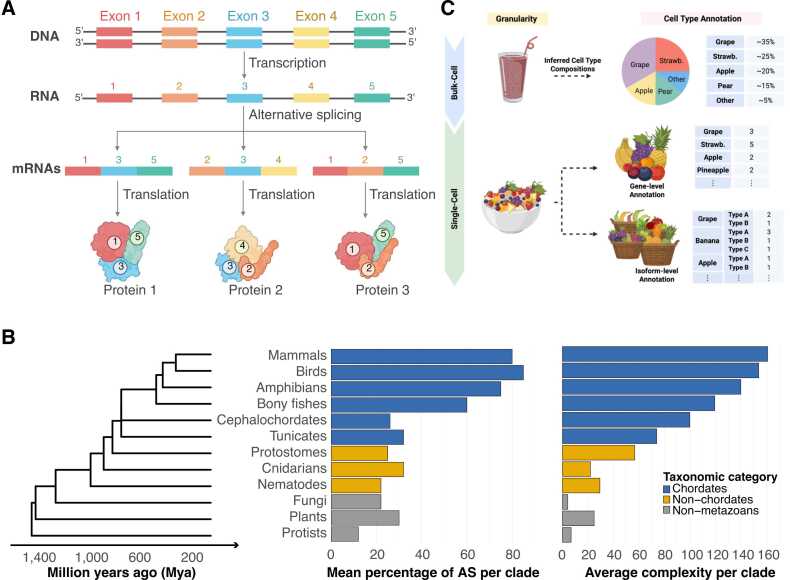
Transcriptomic complexity in evolution and cell type annotation. (A) Process of AS and generation of transcript isoforms. Protein-coding genes are transcribed into precursor mRNA (pre-mRNA) containing exons, introns, and 5′ and 3′ untranslated regions (UTRs). During AS, introns are removed and exons are joined to produce mature mRNA transcripts. The selective inclusion or exclusion of specific exons generates multiple isoforms from a single gene, contributing to proteomic diversity. (B) The phylogenetic tree depicts the evolutionary relationships and divergence timelines of species groups, including mammals, spanning from the present to 1400 million years ago (MYA). The bar charts illustrate the average percentage of alternatively spliced genes for each animal group (middle) and the average complexity of organisms within each taxonomic group (right), quantified by the number of unique cell types as an indicator of organismal complexity. The colours of the bars are categorized based on taxonomic groups (chordates, nonchordate metazoans, or nonmetazoans) [1], [2]. (C) Extension of the fruit salad analogy to illustrate cell type annotation. Bulk RNA sequencing averages gene expression across all cells (smoothie), whereas single-cell RNA sequencing identifies individual cell types (fruit types in the salad). Single-cell isoform sequencing further enhances this resolution by capturing cellular subtypes and fine differences (ripeness or variety of each fruit in the salad).

Alternative splicing (AS) is a fundamental post-transcriptional mechanism that enables a single gene to produce multiple mRNA isoforms by selectively including or excluding specific exons during pre-mRNA processing [1], [76], [77] (Fig. 2A). By allowing genes to produce several transcript isoforms, transcriptomics complexity, protein diversity, and ultimately cellular complexity and their functional versatility can be increased, e.g., in species with fewer protein-coding genes [77], [78], [79], [80].

Isoform diversity rises in proportion to the number of potential combinations of AS events [81] (Fig. 2B). Studies indicate that the fraction of genes that exibit AS has progressively increased over the past 1.4 billion years of eukaryotic evolution, and is significantly correlated with organismal complexity (e.g. quantified by the number of unique cell types; Fig. 2B). Therefore, isoforms resulting from AS should also be a relevant consideration in cell type annotation, which could ultimately yield more profound insights.

### 4.1 Single-cell isoform sequencing

The power of single-cell RNA sequencing (scRNA-seq) to distinguish distinct cell types is often illustrated by the well-known fruit smoothie versus fruit salad analogy: Bulk RNA sequencing is like a smoothie, ie. a blended mix of fruits, because gene expression is averaged across all cell types present, obscuring individual contributions. In contrast, scRNA-seq resembles a fruit salad, where each fruit remains identifiable, as reads are barcoded to indicate their cell of origin, enabling cell-specific gene expression analysis. While gene-level annotation captures the fruit types, single-cell isoform sequencing provides even finer resolution, distinguishing subtle differences such as ripeness or variety, analogous to detecting isoform-level diversity within a cell type (Fig. 2C). In the context of cell-type identification, single-cell isoform sequencing enables the discrimination of cellular subtypes and dynamic states [82], [83], [84], [85], offering an unprecedented level of granularity particularly valuable for tissues with high cell-type diversity.

**Fig. 3:**
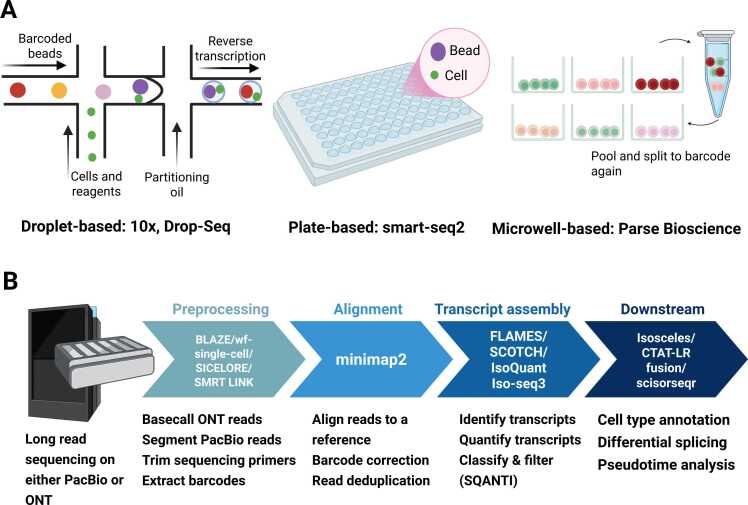
Single-cell Long read RNA-seq workflow. (A) Different methods to barcode single cells include droplet based, plate based, and combinatorial indexing. (B) Basic bioinformatics analysis workflow with examples of tools available for each step.

Single-cell isoform sequencing facilitates the elucidation of cell-specific gene and isoform expression, hence allowing for the identification of biological processes and molecular activities associated with both established and novel cell types. TGS platforms have been tested with single-cell platforms including droplet-, plate-, and microwell-based approaches (Fig. 3A) to advance transcriptome studies by capturing full-length transcripts at single-cell level and increase precision in cellular isoform characterisation. This approach has facilitated applications in human health research, including the identification of novel isoforms, fusion events, and potential neoepitopes for cancer vaccine development [7], [86], [87].

Pacific Bioscience’s (PacBio's) Single-Molecule Real-Time (SMRT) sequencers utilise a silicon chip comprising 8 million (Sequel IIe) or 25 million (Revio) nanometre-scale wells, each acting as a chamber for high-fidelity cDNA sequencing reactions that occur in real time and produce fluorescent signals that must be base-called. Oxford Nanopore Technology (ONT) flowcells contain nanopores embedded in an electro-resistant membrane allowing the identification of change in electric current as nucleotide fragements transfers the pores in real time.

PacBio's *Iso-seq* workflow and ONT's *wf-single-cell* workflow provide end-to-end analysis, from preprocessing raw reads to plotting Uniform Manifold Approximation and Projections (UMAPs). Bioinformatics tools such as *FLAMES* [88] and *SiCeLoRe* [6] implement sequential algorithms to extract barcodes, correct them, assemble transcripts, and generate transcript count matrices. Users can also call variants from their single-cell long-read sequencing data using these tools. After extracting barcodes, reads can be aligned using long-read aligner, such as *minimap2* [89], which retains barcode tags and is widely adopted for splice-aware long-read alignment.

For transcript identification and quantification, tools like *SCOTCH* [90], Isosceles [91] and *IsoQuant* [92] can be used. The resulting isoform count matrix can be used with downstream tools like *Seurat* [93] and *SCANPY* [94] for cell clustering. However, due to a lack of isoform-resolution markers for classification, clustering is typically performed based on gene expression. Isoforms can be classified and filtered using the *SQANTI* [95] workflow to improve cell types annotation (Fig. 3B).

### 4.2 Cell type identification in studies using single-cell long read sequencing

Recently, an increasing number of single-cell long-read sequencing studies have emerged. Here is how they have tackled cell type annotation for their data. Yang et al., (2023) performed parallel single-cell short (Illumina) and long-read sequencing (PacBio) in induced pluripotent stem cell-derived cerebral organoids. After the standard preprocessing of the short-read data and clustering using Seurat, marker genes for each cluster were identified using the FindMarker function [96]. The principal marker genes were then utilised for determining the cell types for each cluster. The long-read data was processed using the PacBio’s Iso-seq3 pipline, followed by isoform annotation using *SQANTI* [95]. However, no refinement of cell type annotations was attempted with the help of the isoform-resolved data.

Dondi et al., (2023) performed single-cell short and long-read sequencing on ovarian cancer patient samples [87]. Cell types in short-read and long-read data were both annotated with *scROSHI* [71], which relies on a priori-defined cell type-specific genes [87]. The high similarity (Jaccard distance) between short and long-read-based cell clusters (exceeding 94 % for most cell types) demonstrated that long-read sequencing aligns well with short-read sequencing for cell type identification. Isoform expression analysis with *SQANTI* [95] revealed an increased transcriptomic diversification in cancer cells indicating that further subclassification of cell types could be achieved via long-read data.

Shiau et al., (2023) developed *scNanoGPS*, a tool that analyses single-cell nanopore sequencing data to capture both cellular genotypes (ie. mutations) and phenotypes (ie. gene isoform expression) for each cell. While the authors performed cell-type-specific isoform analysis, further refinement of cell types based on these patterns was not attempted. In contrast, tumour cells were identified using *CopyKAT*, which inferred chromosomal copy number alteration (CNA) profiles from the UMI count matrix, labelling cells with genome-wide CNAs as tumour cells [97].

In this context, Penter et al., (2024), developed nanoranger, a long-read sequencing workflow that leverages single-cell cDNA libraries to identify cell lineage–defining “natural barcodes,” such as single-nucleotide variants, fusion genes, isoforms, and sequences of chimeric antigen receptors and TCRs [84]. These barcodes are then used for amplicon-based long-read sequencing. Initial cell type annotation is performed using either canonical marker gene expression or alignment of single-cell profiles with a healthy reference dataset. Long-read sequencing then enables the detection of complex variants beyond the reach of short-read sequencing, allowing the tracking of single-cell tumour–immune co-evolution.

Byrne et al., (2024) developed scTaILoR-seq, a targeted long-read sequencing method that enhances isoform detection at single-cell resolution. Applied to ovarian cancer samples, it enabled isoform quantification, variant analysis, and allelic imbalance detection across cell populations. In single-cell data analysis, following preprocessing, cells were clustered using the Leiden algorithm, and the resulting clusters were annotated based on marker gene expression [86].

Li et al., (2024), combined short- and long-read single-cell RNA sequencing to build an isoform-resolution colorectal cancer atlas, identifying dysregulated and tumour-specific isoforms and recurrent neoepitopes for potential cancer vaccines. Cell types were annotated based on gene expression profiles via Seurat’s transfer learning from the Human Colon Cancer Atlas, with tumour epithelial cells refined using Xgboost and copy number variations inferred from the gene expression data. [7].

Collectively, these studies reveal that current approaches to cell type identification in single-cell long-read sequencing still rely heavily on gene-level expression and clustering-based methods. Studies such as Yang et al., (2023), Byrne et al., (2024), Penter et al., (2024), and Li et al., (2024), adopt reference-based annotation strategies, where cell clustering and marker gene identification are performed using tools like Seurat or external reference atlases, while long-read data are primarily used for isoform characterization and validation. Similarly, Dondi et al. [87] and Shiau et al. [97] combine clustering of single-cell profiles with classifiers based on predefined gene signatures or genomic features, such as *scROSHI* and *CopyKAT*, to assign cell identities, maintaining a gene-level focus while exploring isoform diversity within each cell type. Across these studies, clustering remains the foundational step for defining cellular populations, with annotation decisions guided predominantly by marker gene expression rather than isoform-level signatures, highlighting the continued reliance on short-read-based references and the need for computational models that can fully leverage isoform-resolved expression for finer cellular discrimination.

### 4.3 Challenges and opportunities in modelling cell types with single-cell long-read sequencing data

While short-read and long-read sequencing generally produce concordant gene expression estimates for highly expressed genes, this agreement weakens for low-abundance transcripts and rare cell types [98]. Moreover, short-read data often obscure isoform diversity, limiting the ability to distinguish between functionally or developmentally distinct cellular states. The diversity of transcript isoforms, which reflects cell-type–specific splicing programs, introduces a new and biologically meaningful feature space for automated cell type annotation [17], [18].

Long-read sequencing technologies address many of the inherent limitations of short-read methods. In short-read sequencing, RNA molecules are fragmented into short segments (typically ∼150 bp), making it challenging to accurately assign reads to similar isoforms of the same gene [99]. These methods frequently exhibit 3′ bias, insufficient coverage across splice junctions, and difficulty detecting alternative polyadenylation, RNA editing, or fusion transcripts. In contrast, long-read approaches such as PacBio and Oxford Nanopore Technologies (ONT) can capture full-length transcripts in single reads, providing direct insights into complex RNA processing events and isoform coordination. By enabling comprehensive isoform reconstruction, long-read sequencing enhances the resolution of transcriptomic landscapes and offers a powerful foundation for improving the precision and interpretability of automated cell type annotation [17]

Single-cell long-read RNA sequencing generates substantial amounts of data, presenting both opportunities and challenges. Computational techniques must be fast and effective to handle these datasets [31]. ML advancements have enabled the development of fast and accurate computational models [100]. However, high-throughput biological data pose risks of overfitting due to their complexity and small sample sizes, particularly for rare cell types. Additionally, systematic sequencing biases may limit the application of classic learning models [101].

Due to the large dataset size, visualising and interpreting clustering results is challenging. Linear transformation techniques like PCA struggle to capture cellular relationships accurately due to high dropout rates and noise levels. Nonlinear techniques, such as t-Distributed Stochastic Neighbor Embedding (tSNE) and UMAP, offer more flexibility but require careful parameter selections, which significantly impacts visualisation outcomes [20].

The immense number of unannotated isoforms is another major limitation. Undertstanding the splicing mechanisms responsible for transcriptome diversity is crucial for enhancing the precision and efficiency of cell-fate determination modelling as well as refining model assumptions [17]. Since not all genes are relevant for cell type identification, classification models may suffer from overfitting, leading to suboptimal performances [102], [103]. Furthermore, the high frequency of zero reads (dropouts) in single-cell data complicates preprocessing and filtering [20].

Benchmarking of isoform-aware cell-type annotation tools is challenging since there is a lack of datasets combining long-read sequencing with well-curated cell-type labels. One exception is a murine hematopoietic development dataset that provides experimentally validated labels from FACS-isolated embryonic cells [104]. Another potential benchmark dataset is the LongBench cross-platform reference dataset, which was created using 8 cell lines from 3 different cancer types and profiled across 3 long-read sequencing technologies, including ONT, PacBio, and Illumina short-read sequencing. The dataset includes genotype-based cell line annotations [105].

## 5 Discussion

Traditional cell type classification methods heavily depend on prior knowledge and human input. However, since 2018, substantial progress has been made in automated cell type classification technologies. Current models, trained on publicly available single-cell RNA sequencing datasets, can now generate direct predictions of cell types without requiring extensive knowledge of cell markers [19]. This automation has significantly reduced the dependency on manual annotation.

Automatic cell type annotation technologies generally fall into two categories: supervised approaches and prior knowledge-based methods. Prior knowledge-based methods have not consistently outperformed other classifiers, as their effectiveness often hinges on the careful selection of marker genes. While most tools rely on ML methodologies, several non-ML models leveraging statistical techniques have also demonstrated efficacy. The majority of these models have been developed for single-cell RNA sequencing, though a few have been adapted for bulk RNA sequencing data. However, to classify cell types effectively based on isoform expression data, specialised models designed explicitly for long-read RNA sequencing data are essential.

Based on published automatic cell type annotation benchmark studies, traditional classifiers such as SVM, RF, NNs, and Logistic Regression consistently demonstrate strong performance, particularly in well-annotated datasets like Tabula Muris [106] and PBMC3K (www.10xgenomics.com/datasets), where they often achieve F1 scores exceeding 90 %. However, these models struggle in more complex datasets, such as PBMC10K (www.10xgenomics.com/datasets) and snHeart [107], where closely related cell types are more challenging to distinguish.

Among annotation tools, scmap-cell [33], scPred [29], ACTINN [31], and Cell BLAST [36] have performed well in large datasets, while *SingleR* [34], *CP*, and *RPC* [108] have excelled in simulated datasets. However, certain methods, such as *scID* [109] and *Garnett* [110], have underperformed, particularly in self-projection tasks and datasets with heterogeneous cell populations [26]. Notably, while *scVI* [38] has demonstrated strong performance in certain datasets, it has faced challenges in highly annotated datasets such as Tabula Muris and AMB92 (Allan Mouse Brain, http://celltypes.brain-map.org/rnaseq).

Computational efficiency is another critical consideration in cell type annotation. Based on computational time analysis, scmap-cluster [33] has been identified as an efficient model [27]. Many automatic cell type annotation systems incorporate feature selection procedure, which significantly impact model performance. Tools such as *Clustifyr* [111], *SCENIC* [112], and prior knowledge-based methods can be particularly useful for the detection of tumour cells [19].

### 5.1 Emerging trends in cell type annotation

LLMs have emerged as a promising tool for enhancing cell type annotation. By integrating gene expression data with generative AI, LLMs improve the accuracy of identifying rare and complex cell types. Nevertheless, challenges such as data variability and potential inconsistencies presist. Benchmarking studies, such as SOAR [69] and AnnDictionary [13], have demonstrated that LLMs can achieve high accuracy levels (80 – 90 %) in principal cell-type annotations. Emerging tools, such as *LICT* and *GPTCelltype*, offer further advancements in the field.

Owing to technological advancements, particularly in read length, accuracy, and the decreasing cost of sequencing, single-cell long-read RNA sequencing is increasingly being utilised in biological research [84], [95], [96]. This method enables comprehensive molecular profiling and precise annotation of diverse cell types across nearly all tissues of an organism. Accurate identification of cell types is crucial for researchers utilising single-cell long-read sequencing data, as it facilitates a deeper understanding of transcriptome complexity and isoform diversity concerning cell identity, fate, and state transitions [17]. However, two major limitations remain: the relatively high error rate of long-read sequencing compared to short-read sequencing and the uncertainty surrounding the 5′ end of transcripts.

As single-cell long-read RNA sequencing continues to advance, it is imperative to develop specialized annotation models tailored to isoform-level resolution. By addressing current limitations, researchers can enhance the precision and reliability of automated cell type classification, ultimately driving further discoveries in cellular biology and disease research.

The continued advancement of single-cell LLMs will benefit from integrating multi-modal datasets, combining scRNA-seq with isoform-resolved and epigenetic information to capture a more comprehensive view of cellular identity and regulatory mechanisms. Self-supervised pretraining on RNA and single-cell foundation models, leveraging diverse RNA types, including coding, non-coding, and UTR sequences, across multiple organisms, can enhance the ability of models to learn robust, context-aware representations for downstream tasks such as cell type annotation, trajectory inference, and functional prediction [43], [49]. While current LLM applications to isoform-level transcriptomics remain limited, emerging frameworks like *IsoFormer* demonstrate the potential of language-model architectures to integrate multi-modal biological information and capture isoform-level diversity [113]. Complementing tools like *IsoDiffR*, reveals cell-type-specific isoform usage and functional divergence [114].

Training models on continuous-valued, high-dimensional RNA isoform expression matrices poses significant challenges due to the large number of isoforms per gene, sparsity of single-cell measurements, and the need to distinguish isoform-driven from gene-driven expression patterns [82]. Transformer-based approaches such as *IsoFormer* and *IsoDiffR* overcome these challenges by leveraging multi-modal encoders, attention mechanisms, and robust aggregation strategies to capture isoform-specific patterns across cells while integrating complementary DNA, RNA, and protein information [113], [114]. Similarly, these strategies could be extended to isoform-aware LLM architectures, where isoform embeddings and attention-based modeling may enable accurate, context-aware cell type annotation from high-dimensional isoform matrices.

Tokenization and embedding are fundamental steps in adapting LLMs for biological data, enabling the conversion of raw RNA sequences or expression profiles into formats suitable for computational analysis. In RNA sequence data, tokenization methods such as one-hot encoding and k-mer segmentation translate nucleotide strings (A, U, C, G) into numerical representations [49], [115]. For single-cell data, tokenization can be based on gene ranking, expression binning, pathway grouping, or patch-based segmentation of expression matrices [43], [47], [64]. Embedding then maps these tokens into continuous vector spaces, capturing semantic and positional relationships among biological features [49]. Extending these strategies to isoform-level modelling could allow the representation of transcript variants and splicing events, creating isoform-aware embeddings that capture transcript-specific regulatory information. While current models primarily rely on gene-aware embeddings to represent cell states, the development of isoform-level tokenization and embeddings remains an open and promising research direction for improving automated cell type annotation and understanding splicing-driven cellular diversity.

Additionally, explainable AI plays a crucial role in validating LLM-based biological predictions by linking model outputs to interpretable biological features such as genes, pathways, or isoforms. By making tokenization and embedding steps biologically transparent, explainable AI enables researchers to trace how specific molecular patterns influence model decisions, thereby increasing trust and interpretability in LLM-driven single-cell analyses [49].

## Authors' contributions

HW surveyed the literature and wrote the first draft. SB provided substantial technical advice on single-cell isoform sequencing technologies. NB and DS contributed paragraphs on NLP and LLMs. SW and US supervised the work, supported data visualisation, and helped with reviewing and revising the manuscript. All authors read and approved the final manuscript.

## CRediT authorship contribution statement

**Debarka Sengupta:** Writing – review & editing, Supervision. **Siyuan Wu:** Writing – review & editing, Supervision, Conceptualization. **Ulf Schmitz:** Writing – review & editing, Writing – original draft, Supervision, Funding acquisition, Conceptualization. **Hettiarachchige Wijewardena:** Writing – review & editing, Writing – original draft, Investigation, Formal analysis. **Saloni Bhatia:** Writing – original draft, Visualization, Investigation. **Namrata Bhattacharya:** Writing – original draft, Conceptualization.

## Funding

This work was supported by the 10.13039/501100000925National Health and Medical Research Council (Grant #1196405 to US); the Tropical Australian Academic Health Centre (Grant #SF01124); the Townsville University Hospital (Grant #THHSSERTA_RPG05_2024, THHSSERTA_RPG15_2024, and THHSSERTA_RCG05_2024). HW is supported by a James Cook University International Higher Degree Research Fellowship.

## Declaration of Competing Interest

The authors declare that they have no known competing financial interests or personal relationships that could have appeared to influence the work reported in this paper.
