# Supplementary material for: Advancing automated cell type annotation with large language models and single-cell isoform sequencing
Source: Comput Struct Biotechnol J. 2025 Nov 6;27:4952–62. doi: 10.1016/j.csbj.2025.11.008 (PMC12657604; doi:10.1016/j.csbj.2025.11.008)
Supplement: Supplementary file 1 — Supplementary material [file mmc1.docx]

## Supplementary Materials

Supplementary Table 1: Automated cell type annotation tools for RNA sequencing data, implemented between 2014 and 2024. The 'Unassigned' function represents whether the tool assigns cells that are uncertain in classification, such as those not meeting the predefined threshold of the respective tool. Tools without any symbol indicate those that work exclusively with scRNA-seq data, '+' denotes tools that utilise both scRNA-seq and bulk RNA-seq data, '-' indicates tools that utilise only bulk RNA-seq data, and '*' represents tool that utilised microarray data. UMI - Unique Molecular Identifier; FPKM - Fragments Per Kilobase of transcript per Million mapped reads; TPM - Transcripts Per Million; RBF - Radial Basis Function, KNN - K-Nearest Neighbors; XGBoost - Extreme Gradient Boosting.

| **Name of the tool** | | **Classifier/Method/ Computational approach** | | | **Ability to predict new cell types** | | | **Unassigned function** | | | **Input format** | | | **Language** | | | **Reference** | |  |  |
| --- | --- | --- | --- | --- | --- | --- | --- | --- | --- | --- | --- | --- | --- | --- | --- | --- | --- | --- | --- | --- |
| **Neural Network Approaches** | | | | | | | | | | | | | | | | | | |  |  |
| + | *ACTINN* | | | Neural Network | | | Yes | | | Yes | | | Count matrix | | | Python | | (1) | | |
|  | *Cell BLAST* | | | Neural Network | | | Yes | | | Yes | | | Count matrix | | | Python | | (2) | | |
|  | *CellTICS* | | | Neural Network | | | No | | | No | | | Count matrix | | | Python | | (3) | | |
|  | *Deep learning-based single-cell type prediction tool* | | | Neural Network | | | No | | | No | | | Gene expression matrix | | | Python | | (4) | | |
|  | *JIND* | | | Neural Network | | | Yes | | | Yes | | | UMI counts, FPKM, or TPM | | | Python | | (5) | | |
|  | *Kratos* | | | Neural Network | | | No | | | No | | | Gene expression profile | | | Python | | (6) | | |
|  | *LAmbDA* | | | Feedforward Neural Network with bagging | | | No | | | Yes | | | Gene expression profile | | | Python | | (7) | | |
|  | *MapCell* | | | Siamese Neural Networks | | | Yes | | | No | | | Gene expression profile | | | R | | (8) | | |
|  | *MarkerCapsule* | | | Capsule network | | | No | | | No | | | Gene expression profile | | | Python | | (9) | | |
|  | *MARS* | | | Neural Network | | | Yes | | | No | | | Gene expression profile | | | Python | | (10) | | |
|  | *Matilda* | | | Neural Network | | | Yes | | | No | | | Count matrix | | | Python | | (11) | | |
|  | *mtANN* | | | Ensemble of neural network-based deep classifiers | | | Yes | | | Yes | | | Gene expression profile | | | Python (R support for Bioinformatics) | | (12) | | |
|  | *OnClass* | | | Bilinear Neural Network | | | Yes | | | Yes | | | Count matrix | | | Python | | (13) | | |
|  | *scBERT* | | | Deep Neural Network | | | Yes | | | Yes | | | Gene expression profile | | | Python | | (14) | | |
|  | *scCapsNet* | | | Capsule network | | | Yes | | | Yes | | | Gene expression profile | | | Python | | (15) | | |
|  | *scDeepSort* | | | Weighted graph neural network | | | No | | | Yes | | | Count matrix | | | python | | (16) | | |
|  | *scNym* | | | Neural Network | | | Yes | | | Yes | | | Gene expression matrix | | | Python | | (17) | | |
|  | *scPretrain* | | | Neural Network | | | No | | | No | | | Gene expression matrix | | | Python | | (18) | | |
|  | *scSemiGCN* | | | Graph Convolutional Networks | | | No | | | No | | | scRNA-seq expression matrix | | | Python | | (19) | | |
|  | *scTab* | | | Neural Network | | | No | | | Yes | | | Gene expression matrix | | | Python | | (20) | | |
|  | *scVI* | | | Stochastic optimization and deep neural networks | | | No | | | No | | | Raw count matrix | | | Python | | (21) | | |
|  | *SigPrimedNet* | | Artificial Neural Network | | | Yes | | | Yes | | | Count matrix | | | Python | | | (22) | |  |
|  | *SuperCT* | | Artificial Neural Network | | | Yes | | | Yes | | | Binary gene expression data | | | Python | | | (23) | |  |
|  | *TripletCell* | | Deep metric learning-based algorithm | | | Yes | | | Yes | | | Gene expression matrix | | | Python | | | (24) | |  |
| **Support Vector Machine (SVM) Approaches** | | | | | | | | | | | | | | | | | | |  |  |
| + | *Besca* | | Signature-based hierarchical cell annotation or support Vector Machines | | | No | | | No | | | Count matrix | | | Python | | | (25) | |  |
| - | *CIBERSORT* | | Nu-support vector regression | | | No | | | No | | | Gene expression profiles | | | Java and R | | | (26) | |  |
|  |  | |  | | |  | | |  | | |  | | |  | | |  | |  |
|  | *scCD45RA* | | Support Vector Machine with an RBF kernel | | | No | | | No | | | Gene expression profile | | | Python | | | (27) | |  |
|  | *scClassifR* | | Support Vector Machine | | | No | | | Yes | | | Seurat objects(From Seurat) or SingleCellExperiment objects (from the Bioconductor package) | | | R | | | (28) | |  |
|  | *scHPL* | | Linear Support Vector Machine , kNN, or one-class Support Vector Machine | | | Yes | | | Yes | | | Gene expression profile | | | Python | | | (29) | |  |
|  | *scPred* | | Support Vector Machine | | | No | | | Yes | | | Gene expression matrix | | | R | | | (30) | |  |
| **Tree & Ensemble Learning Approaches** | | | | | | | | | | | | | | | | | | |  |  |
|  | *CaSTLe* | | XGBoost | | | No | | | Yes | | | Count matrix | | | R | | | (31) | |  |
|  | *CellHint* | | Predictive clustering  tree | | | No | | | Yes | | | Gene expression matrix | | | Python | | | (32) | |  |
| * | *CellNet* | | Random Forest | | | No | | | No | | | Gene expression profiles | | | Java | | | (33) | |  |
|  | *CTISL* | | Support Vector Machine and logistic regression | | | No | | | No | | | Count matrix | | | Python | | | (34) | |  |
|  | *HieRFIT* | | Hierarchical Random Forest | | | No | | | Yes | | | Gene expression matrix | | | R | | | (35) | |  |
|  | *Immune cell type signature* | | Random Forest | | | No | | | No | | | Gene expression profile | | | R | | | (36) | |  |
|  | *MACA* | | Normalized confusion matrix and ensembled annotation | | | No | | | Yes | | | Count matrix | | | Python | | | (37) | |  |
|  | *NS-Forest* | | Random Forest | | | No | | | Yes | | | Count matrix | | | Python | | | (38) | |  |
|  | *scAnnotate* | | Supervised ensemble machine learning | | | No | | | No | | | Count matrix | | | R | | | (39) | |  |
|  | *scClassify* | | Ensemble learning | | | Yes | | | Yes | | | Log-transformed gene expression matrix | | | R | | | (40) | |  |
|  | *SCDC* | | Bootstrap resampling | | | No | | | No | | | Count matrix | | | R | | | (41) | |  |
|  | *scDetect* | | Weighted ensemble k-TSP algorithm | | | No | | | Yes | | | Count matrix | | | R | | | (42) | |  |
|  | *scIAE* | | Ensemble classification framework based on integrative autoencoders | | | No | | | Yes | | |  | | | R | | | (43) | |  |
|  | *SHARP* | | Ensemble random projection-based | | | No | | | No | | | Gene expression matrix | | | R | | | (44) | |  |
|  | *SingleCellNet* | | Random Forest | | | Yes | | | Yes | | | Count matrix | | | R and Python | | | (45) | |  |
|  | *Superscan* | | XGBoost | | | Yes | | | Yes | | | Raw count matrix | | | Python | | | (46) | |  |
| **Dimensionality Reduction and Clustering Approaches** | | | | | | | | | | | | | | | | | | |  |  |
| + | *CellAtlasSearch* | | Locality-Sensitive Hashing | | | Yes | | | No | | | Count matrix | | | - | | | (47) | |  |
|  | *CellFishing.jl* | | Cosine similarity of Hamming locality sensitive hashing | | | Yes | | | No | | | Digital gene expression (DGE) matrix | | | Julia | | | (48) | |  |
|  | *DUSC* | | K-means (KM) and expectation maximization | | | Yes | | | No | | | Count matrix | | | Python | | | (49) | |  |
|  | *Northstar* | | k nearest neighbors graph and Leiden algorithm | | | Yes | | | Yes | | | Count matrix | | | Python | | | (50) | |  |
|  | *scANVI* | | KNN Classifier | | | Yes | | | Yes | | | Count matrix | | | Python | | | (51) | |  |
|  | *SCINA* | | Expectation-maximization algorithm | | | No | | | Yes | | | Gene expression matrix | | | R | | | (52) | |  |
|  | *scmap-cell* | | KNN classification with cosine similarity | | | No | | | Yes | | | Count matrix | | | R | | | (53) | |  |
|  | *scmap-cluster* | | Similarities between each cell and all centroids of the reference data set | | | No | | | Yes | | | Count matrix | | | R | | | (53) | |  |
| **Logistic Regression & Regression-Based Approaches** | | | | | | | | | | | | | | | | | | |  |  |
| + | *CellO* | | | Logistic Regression and Correction Techniques | | | No | | | No | | | Gene expression profiles | | | Python | | (54) | | |
|  | *CellTypist* | | | Logistic Regression | | | No | | | No | | | Count matrix | | | Python | | (55) | | |
|  | *Garnett* | | | Hierarchical cell type annotation using an elastic-net regularized multinomial generalized linear model (GLMnet) | | | No | | | Yes | | | Gene expression matrix | | | R | | (56) | | |
|  | *ImmClass2019* | | | Elastic-net logistic regression | | | No | | | No | | | Count matrix | | | R | | (57) | | |
| - | *Imply* | | | Support Vector Regression, Linear Mixed Effect Model, Non-Negative Least Squares | | | No | | | No | | | Gene expression profile | | | R | | (58) | | |
|  | *IsoDeconvMM* | | | Maximum likelihood | | | No | | | No | | | RNA-seq data (read counts at the exon set level) | | | R | | (59) | | |
|  | *SciBet* | | | Maximum likelihood estimation | | | Yes | | | Yes | | | Gene expression profile | | | R | | (60) | | |
|  | *UNIFAN* | | | Logistic Regression | | | No | | | No | | | AnnData files containing scRNA-seq gene expression data | | | Python | | (61) | | |
| **Template & Prototype-Based Approaches** | | | | | | | | | | | | | | | | | | |  |  |
|  | *MarkerCount* | | | Gene-cell type association score | | | Yes | | | Yes | | | Binarized gene expression matrix | | | Python | | (62) | | |
|  | *scEVOLVE* | | | Prototype-parameterized classifier | | | Yes | | | No | | | Gene expression matrix | | | Python | | (63) | | |
|  | *scTyper* | | | Nearest Template Prediction, Gene Set Enrichment Analysis, and average expression values | | | No | | | No | | | FASTQ files or Seurat objects | | | R | | (64) | | |
| **Hierarchical & Rule-Based Approaches** | | | | | | | | | | | | | | | | | | |  |  |
|  | *CellAssign* | | | Hierarchical statistical framework | | | Yes | | | Yes | | | Gene expression matrix | | | R | | (65) | | |
|  | *CHETAH* | | | Hierarchical classification tree based on Spearman correlation | | | Yes | | | Yes | | | Count matrix | | | R | | (66) | | |
|  | *Moana* | | | Hierarchical classification, KNN-smoothing and Support Vector Machine | | | No | | | Yes | | | Gene expression profile | | | Python | | (67) | | |
|  | *StemID* | | | Lineage tree | | | Yes | | | No | | | Gene expression matrix | | | R | | (68) | | |
| **Statistical & Correlation-Based Approaches** | | | | | | | | | | | | | | | | | | |  |  |
| + | *Clustifyr* | | | Spearman correlation | | | Yes | | | Yes | | | Count matrix | | | R | | (69) | | |
| + | *deCS* | | | Highest Pearson correlation coefficient or Fisher's exact test and highest intersection ratio. | | | Yes | | | Yes | | | Expression profile or marker genes of a cell cluster | | | R | | (70) | | |
|  | *scCATCH* | | | Evidence-based scoring | | | No | | | No | | | Count matrix | | | R | | (71) | | |
|  | *scID* | | | Fisher’s linear discriminant analysis | | | No | | | Yes | | | Gene expression profile | | | R | | (72) | | |
|  | *scMatch* | | | Spearman and Pearson correlations | | | No | | | No | | | Gene expression matrix | | | Python | | (73) | | |
|  | *scMayoMap* | | | Marker gene scoring and reference database matching | | | No | | | No | | | Cluster marker gene list | | | R | | (74) | | |
|  | *scMCA* | | | Pearson correlation coefficient | | | NO | | | No | | | DGE matrix | | | R | | (75) | | |
|  | *scROSHI* | | | One-sided Mann-Whitney test with score-based decision rules | | | Yes | | | Yes | | | Count matrix | | | R | | (76) | | |
|  | *SCSA* | | | Score annotation model | | | Yes | | | No | | | Differentially expressed genes clusters matrix | | | Python | | (77) | | |
| + | *SingleR* | | | Spearman correlation | | | No | | | No | | | Gene expression matrix | | | R | | (78) | | |
| **Transfer Learning & Other Machine Learning Approaches** | | | | | | | | | | | | | | | | | | |  |  |
|  | *CIForm* | | | Transformer based approach | | | N0 | | | No | | | Gene expression matrix | | | Python | | (79) | | |
|  | *ItClust* | | | Transfer learning and Kullback-Leibler divergence between distribution P and Q | | | Yes | | | Yes | | | UMI count, FPKM, or TPM | | | Python | | (80) | | |
|  | *RMTL* | | | Regularized multi-task learning | | | Yes | | | No | | | Count matrix | | | R | | (81) | | |
|  | *SCENIC* | | | Latent Dirichlet Allocation, Gradient Boosting Machine Regression and Motif enrichment analysis | | | No | | | No | | | Count matrix | | | Python | | (82) | | |
|  | *scGAD* | | | Anchor-basedself-supervised learning module | | | Yes | | | Yes | | | Gene expression profile | | | Python | | (83) | | |
|  | *scLearn* | | | Metric learning-based model | | | Yes | | | Yes | | | Gene expression matrix | | | R | | (84) | | |
|  | *scTransSort* | | | Transformer based approach | | | No | | | No | | | Gene expression matrix | | | Python | | (85) | | |
|  | *Seurat* | | | Transfer learning | | | No | | | No | | | Count matrix | | | R | | (86) | | |
|  | *TOSICA* | | | Transformer-based approach | | | Yes | | | Yes | | | Gene expression matrix | | | Python | | (87) | | |
|  | *TransCluster* | | | Transformer and CNN | | | No | | | No | | | Gene expression matrix | | | Python | | (88) | | |
| **Hybrid Machine Learning Approaches** | | | | | | | | | | | | | | | | | | |  |  |
|  | *DigitalCellSorter* | | | Voting algorithm and a Hopfield classifier | | | Yes | | | Yes | | | Count matrix | | | Python | | (89) | | |
|  | *ImmClassifier* | | | Random Forest classifier and Deep Neural Network | | | No | | | No | | | Gene expression profile | | | R | | (90) | | |
|  | *scASK* | | | Data Adaptive Slicing, Meta Classifiers Selecting and Ensemble Mode Switching | | | No | | | No | | | Gene expression profile | | | Matlab | | (91) | | |
|  | *scReClassify* | | | Support Vector Machine and Random Forest | | | No | | | No | | | Normalized gene expression profiles | | | R | | (92) | | |
|  | **Marker based approach** | | | | | | | | | | | | | | | | | | | |
|  | *Sargent* | | | Marker gene scoring with Gini-based trimming and optional smoothing | | | No | | | Yes | | | Gene expression matrix and list of cell type gene markers | | | R | | (93) | | |
|  | *ScType* | | | Rule-based, marker-gene scoring method | | | No | | | Yes | | | Raw or pre-processed/normalized expression matrices | | | R | | (94) | | |

Supplementary Table 2: Datasets utilised in Automatic cell type annotation tools benchmark studies. PBMC- Peripheral Blood Mononuclear Cells; scHeart- Single cell RNA seq Heart dataset; snHeart- Single-nucleus RNA-seq ; FACS- Fluorescence-Activated Cell Sorting; InDrop: Indexing droplet.

| **Dataset** | **Description** | | **Sequencing protocol/ technology** | **Cell count** | **Dataset reference** | **Benchmark** |
| --- | --- | --- | --- | --- | --- | --- |
| **Human** | | | | | | |
| PBMC_10Xv2 | | PBMC | 10x Genomics | 6444 | https://portals.broadinstitute.org/single_cell/study/SCP424/single-cellcomparisonpbmc-data | Huang and Zhang |
| PBMC_10Xv3 | | PBMC | 10x Genomics | 3222 |  |  |
| Baron Human | | Pancreas | InDrop | 8562 | https://hemberg-lab.github.io/scRNA.seq.datasets/human/pancreas/ |  |
| Muraro | | Pancreas | CEL-Seq2 | 2119 |  |  |
| Segerstolpe | | Pancreas | SMART-Seq2 | 2111 |  |  |
| Human normal lung data | | Lung | Illumina HiSeq 2500 | 208506 | (95) | Xie |
| PBMC_Sorted | | PBMC | 10x Genomics | 94655 | (96) | Zhao |
| Baron Human | | Pancreas | InDrop | 8569 | (97) |  |
| Muraro | | Pancreas | CEL-Seq2 | 2122 | (98) |  |
| Xin | | Pancreas | SMARTer | 1492 | (99) |  |
| Baron Human | | Pancreas | InDrop | 8569 | (97) | Abdelaal |
| Muraro | | Pancreas | CEL-Seq2 | 2122 | (98) |  |
| Segerstolpe | | Pancreas | SMART-Seq2 | 2133 | (100) |  |
| Xin | | Pancreas | SMARTer | 1449 | (99) |  |
| CellBench 10X | | A mixture of five human lung cancer cell lines | 10x Genomics | 3803 | (101) |  |
| PBMC_Sorted | | PBMC | 10x Genomics | 91649 | (96) | Huang |
| PBMC3K | | PBMC | 10x Genomics | 2467 | https://support.10xgenomics.com/single-cell-gene-expression/datasets |  |
| Pancreas CEL-Seq2 data | | Pancreas | CEL-Seq2 | 2285 | (102) |  |
| Pancreas Fluidigm C1 data | | Pancreas | Fluidigm C1 | 638 | (102) |  |
| PBMC Zheng | | PBMC | 10x Genomics | 61309 | (96) | Sun |
| **Mouse** | | | | | | |
| Tabula Muris lung data | | Lung | 10x Genomics, SMART-Seq2 | 5449 | https://tabula-muris.ds.czbiohub.org/ | Xie |
| Baron Mouse | | Mouse pancreas | InDrop | 1886 | (97) | Abdelaal |
| Tabula Muris | | Whole Mus musculus | SMART-Seq2 | 54865 | https://tabula-muris.ds.czbiohub.  org/ |  |
| Allen Mouse Brain | | Primary mouse visual cortex | SMART-Seq v4 | 12832 | http://celltypes.brain-map.org/rnaseq |  |
| Zheng sorted | | FACS-sorted PBMC | 10x Genomics | 20000 | https://support.1  0xgenomics.com/single-cell-gene-expression/datasets; |  |
| Zheng 68Ka | | PBMC | 10x Genomics | 65943 | https://support.10xgenomics.com/  single-cell-gene-expression/datasets |  |
| Allen Mouse Brain | | AMB3 | SMART-Seq | 12826 | http://celltypes.brain-map.o  rg/rnaseq | Huang and Zhang |
| Allen Mouse Brain | | AMB16 | SMART-Seq | 12811 |  |  |
| Allen Mouse Brain | | AMB93 | SMART-Seq | 12781 |  |  |
| Tabula Muris | | Whole Mus musculus | SMART-Seq2 | 54865 | https://tabu  la-muris.ds.czbiohub.org |  |
| Anderson | | Brain Striatum | 10x Genomics | 14466 | (103) | Lakkis |
| Jessa.Pons | | Pons | 10x Genomics | 25978 | (104) |  |
| Vladoiu | | Cerebellum | 10x Genomics | 62040 | (105) |  |
| Jessa.Brain | | Brain | 10x Genomics | 58153 | (104) |  |
| Tabula Muris full SMART-Seq2 data | | Whole Mus musculus | SMART-Seq2 | 24622 | (106) | Huang |
| Tabula Muris full 10x Genomics | | Whole Mus musculus | 10x Genomics | 20000 |  |  |
| Tabula Muris lung SMART-Seq2 data | | Lung | SMART-Seq2 | 1563 |  |  |
| Tabula Muris lung 10x Genomics | | Lung | 10x Genomics | 1303 |  |  |
| PBMC3K | | PBMC | 10x Genomics | 2664 | http://www.10xgenomics.com/datasets | Tortelote |
| PBMC10K | | PBMC | 10x Genomics | 9765 |  |  |
| scHeart | | Heart | Illumina NextSeq 500 | 4000 | (107) |  |
| snHeart | | Heart | Single-nucleus RNA seq dataset | 4000 |  |  |
| **Human and Mouse** | | | | | | |
| Mix4 | | K562, HEK293T  A431, and L929 cell lines | Illumina HiSeq X | 1101 | (108) | Zhao |
| Mix3 | | K562, HEK293T  and L929 cell lines | Illumina HiSeq X | 2215 |  |  |
| **Simulates datasets** | | | | | | |
| Simulated true assay without dropouts | | A mixture of five cell types | *Splatter* (R package) | 2000 | (109) | Huang |
| Simulated raw assay with dropout mask | | A mixture of five cell types | *Splatter* (R package) | 2000 |  |  |

Supplementary Table 3: Detailed information on each LLM-based cell type annotation tool.

| **Tool** | **Input Modalities** | **Training Data Source** | **Training Data Size** | **Model Interpretability** | **Computational Cost** | **Biological Validation Metrics** |
| --- | --- | --- | --- | --- | --- | --- |
| **LICT** (Large Language Model–based Identifier for Cell Types) (110) | Single-cell RNA-seq data | Multiple scRNA-seq datasets (PBMC, gastric cancer, human embryo, fibroblast, stromal) | – | Interpretable through the *“talk-to-machine”* framework, enabling objective evaluation and self-assessment of annotation reliability | Not explicitly reported | Annotations generated by LLMs were directly compared to manual annotations |
| **GPTCelltype** (111) | Marker genes or top differentially expressed genes | Pretrained LLMs (GPT-3.5 and GPT-4) via OpenAI API | – | Limited transparency due to undisclosed GPT-4 training data; human evaluation required for quality assurance | No inference cost (retrained model) but involves financial cost for API use | Agreement with manual annotations from original studies |
| **CellAgent** (112) | Single-cell RNA-seq data, natural language task descriptions | GPT-4/4V pretrained models | – | Partial transparency via biologically specialized agents (*Planner, Executor, Evaluator*); employs hierarchical task decomposition | No training cost; moderate inference cost due to multi-agent execution | Compared predicted labels against expert-annotated datasets |
| **CASSIA** (113) | scRNA-seq data, species, tissue type, and marker genes | Pretrained models: Claude 3.5 Sonnet, GPT-4o, LLaMA-3.2 90B | 970 cell types | Provides detailed reasoning with validator cross-checks, quantitative quality scores, and HTML reports documenting each decision | About 30 s per cell type per CPU core; about 2 min for 20 clusters on an 8-core system | Validated through iterative feedback between Annotator and Validator agents ensuring marker consistency |
| **Celler** (114) | Single-cell gene expression matrix | Celler-75 dataset (40 M cells from 80 human tissues and 75 diseases) and public datasets (MS, hPancreas) | Celler-75: 41.3 M cells; MS: 13,468; Pancreas: 10,600 | Provides prediction confidence scores as model output | Not explicitly reported | Accuracy, Precision, and Recall evaluated against fine-grained functional and marker-based cell types |
| **CellReasoner** (115) | Natural language–style “cell sentences” from top highly variable genes (HVGs) | Expert-annotated pan-cancer scRNA-seq datasets | 37,187 cells (1,000 cells × 387 per type) | Employs reasoning-augmented annotation with chain-of-thought (CoT) supervision combining AI- and human-curated reasoning paths | Single A800 GPU; LoRA fine-tuning; batch 8–20; 8,192 max tokens | Accuracy evaluated via direct and CoT tasks; validated against marker genes and reasoning consistency |
| **CellTypeAgent** (116) | Marker gene lists (per tissue/species) | CellxGene database | 41 M cells across 714 cell types | LLM reasoning combined with gene expression verification | Inference cost increases with prompt length | Accuracy assessed using manual annotations from benchmark studies |
| **ReCellTy** (117) | Cell identifiers and associated marker/top differential genes | CellMarker 2.0 database | 1,528 naming features; 61,049 gene–cell type associations | Provides intermediate reasoning steps and feature selection with transparent annotation tracing | Dependent on query size; processed using GraphRAG with Cypher queries | Validated via manual evaluation and semantic similarity using text-embedding cosine scores |
| **scBERT** (14) | scRNA-seq gene expression matrices | Public scRNA-seq data from PanglaoDB | Over 1 M cells | Uses attention mechanism to highlight gene contributions for each cell type; supports scalable | Performer attention enables efficient full-gene representation processing of over 16,000 genes | Demonstrated biological relevance by highlighting key genes linked to known and novel markers |
| **scExtract** (118) | PDF articles and corresponding raw count data (h5ad) | Curated single-cell skin datasets (14 publications via NCBI) | 440,000 cells | Provides confidence scores and logs explaining annotation decisions | Scalable to about 1 M cells (less than 20 min processing; less than $1 via API) | Predictions compared with manual annotations for accuracy assessment |
| **scInterpreter** (119) | scRNA-seq expression data and descriptive text for each gene extracted from NCBI dataset | HUMAN-10k and MOUSE-13k scRNA-seq datasets | HUMAN-10k: 10k cells (61 types); MOUSE-13k: 13k cells (37 types) | Frozen LLM ensures stable reasoning; interpretable class-token and classification head enable transparent mapping | Not explicitly reported; GPT-3.5 (for gene embeddings), LLaMA-13B (for classification) | Evaluated using Accuracy, Precision, Recall, and F1 scores |
| **scGPT** (120, 121) | Annotated scRNA-seq data (AnnData h5ad format) | Retina snRNA-seq dataset | 2.9 M cells; 111 cell types | Interpretability evaluated via ablation studies identifying genes most influential for classification | Tested on NVIDIA A100/A30/RTX A5000; batch size 32; high GPU usage | Evaluated using Accuracy, Precision, Recall, F1-score, and Cohen’s Kappa |

Supplementary Table 4: Summary of datasets and associated cell types used to evaluate the annotation accuracy of large language model (LLM)-based cell type annotation frameworks.

| **Dataset** | **Description/Cell types** | | **Dataset reference** | **Tool** |
| --- | --- | --- | --- | --- |
| Human peripheral blood mononuclear cells | | Human PBMCs | (122) | LICT (110) |
| Fibroblasts from various organs | | Fibroblasts | (123) |  |
| Gastric tumour samples | | Tumour and matched normal tissue from gastric (stomach) tissue human | (124) |  |
| Human embryo data | | Mouse Normal | (125) |  |
| Mouse Cell Atlas (MCA) | | Human Normal/Cancer | (126) | GPTCelltype (111) |
| Human Cell Landscape (HCL) | | Human Cancer | (127) |  |
| Lung cancer | | Human Cancer | (128) |  |
| Colon cancer | | Human colorectal cancer, | (129) |  |
| Azimuth | | Human Normal | https:  //azimuth.hubmapconsortium.org/ |  |
| Non-model mammal | | Cat, tiger, and pangolin NA | (130) |  |
| GTEx | | Human Normal | (131) |  |
| literature (from GTEx) | | Human NA | (131) |  |
| Tabula Sapiens (TS) | | Human Normal | (132) |  |
| B cell lymphoma (BCL) | | Human Cancer | (133) |  |
| PBMC dataset | | PBMCs | https://www.10xgenomics.com/resources/  datasets/8-k-pbm-cs-from-a-healthy-donor-2-standard-2-1-0  (96) | CellAgent (112) |
| Pancreas dataset | | The pancreas datasets comprise Baron, Muraro,  Segerstolpe and Xin | https://hemberg-lab.  github.io/scRNA.seq.datasets/human/pancreas/ |  |
| Multiple PBMC datasets | | PBMCs | accession code is SCP424 |  |
| Immune dataset from different donors | | Human immune cells | https://figshare.com/ndownloader/files/25717328 |  |
| mouse dataset from the brain region | | Mouse brain cells | https://portal.brain-map.org/atlases-and-data/  rnaseq/mouse-whole-cortex-and-hippocampus-10x |  |
| GTEx | | Cells across multiple human tissues | (131) | CASSIA (113) |
| Tabula Sapiens (TS) | | 24 human tissues, capturing over 400 cell types | (132) |  |
| Human Cell Landscape (HCL) | | Stem/progenitor cells, differentiated cell types | (127) |  |
| Mouse Cell Atlas (MCA) | | Cells covering all of the major mouse organs | (126) |  |
| Azimuth | | Major cell types across multiple tissues, including immune, epithelial, endothelial, stromal, and stem/progenitor cells | https://azimuth.hubmapconsortium.org/ |  |
| Celler-75 | | 75 specific human disease |  | Celler (114) |
| MS | | Multiple Sclerosis | (134) |  |
| hPancreas | | The pancreas datasets comprise Baron, Muraro, Lawlor,  Segerstolpe and Xin | (97–100, 102) |  |
| Pancreatic Ductal Adenocarcinoma comprises (PDAC) dataset | | Pancreas-Related  [Homo sapiens](https://www.ncbi.nlm.nih.gov/Taxonomy/Browser/wwwtax.cgi?mode=Info&id=9606) | https://www.ncbi.nlm.nih.gov/geo/query/acc.cgi?acc=GSE197177 | CellReasoner (115) |
| PBMC3K dataset | | Healthy human donor peripheral blood 499 mononuclear cells | https://www.10xgenomics.com/cn/datasets/3-k-pbm-cs-from-a-healthy-donor-1-standard-1-1-0 |  |
| Multiome PBMC dataset | | Human PBMCs | https://support.10xgenomics.com/single-cell-multiome-atac-gex/datasets/1.0.0/pbmc_granulocyte_sorted_10k |  |
| Liver dataset | | [Homo sapiens](https://www.ncbi.nlm.nih.gov/Taxonomy/Browser/wwwtax.cgi?mode=Info&id=9606) liver | https://www.ncbi.nlm.nih.gov/geo/query/acc.cgi?acc=GSE115469 |  |
| Pancancer38k | | Pan-cancer single-cell studies | aggregated from multiple published pan-cancer single-cell studies |  |
| Mouse Cell Atlas (MCA) | | cells covering all of the major mouse organs | (126) | CellTypeAgent (116) |
| Human Cell Landscape (HCL) | | Stem/progenitor cells, differentiated cell types | (127) |  |
| Lung cancer | | Human Cancer | (128) |  |
| Colon cancer | | Human colorectal cancer, | (129) |  |
| Azimuth | | Major cell types across multiple tissues, including immune, epithelial, endothelial, stromal, and stem/progenitor cells | https:  //azimuth.hubmapconsortium.org/ |  |
| Non-model mammal | | Non-model species, including cat, dog, hamster, lizard, goat, rabbit, duck, pigeon, pangolin, tiger, and deer, covering lung cell types | (130) |  |
| GTEx | | Cells across multiple human tissues | (131) |  |
| Tabula Sapiens (TS) | | 24 human tissues, capturing over 400 cell types | (132) |  |
| B cell lymphoma (BCL) | | Human Cancer | (133) |  |
| Cell-  Marker 2.0 | | Human and mouse cell types across 656 tissues | http:  //www.bio-bigdata.center/CellMarker download.  html, | ReCellTy (117) |
| Azimuth | | Major cell types across multiple tissues, including immune, epithelial, endothelial, stromal, and stem/progenitor cells | https://azimuth.hubmapconsortium.org/ |  |
| The Panglao dataset | | 209 human single-cell datasets | https://panglaodb.se/ | scBERT (14) |
| Zheng68k dataset | | PBMC cells | (96) |  |
| Pancreas datasets | | The pancreas datasets comprise Baron, Muraro,  Segerstolpe and Xin | (97–100) |  |
| MacParland dataset | | 20 hepatic cell populations | (135) |  |
| Heart datasets: Pretraining | | Human heart cells | (136) |  |
| Heart datasets: benchmarking | | Human heart cells | (137) |  |
| The lung dataset for COVID-19 study | | Human lung tissue and analysed  for COVID-19-related disease mechanisms | (138) |  |
| Human Cell Atlas dataset | | Human cells across 15 organs | (139) |  |
| Skin autoimmune disease dataset | | Various skin cell types across diseases including psoriasis, atopic dermatitis (AD), acne, granuloma annulare (GA); neonates to elderly | (140) | scExtract (118) |
| Pancreas dataset | | Pancreatic cells | <https://github.com/theislab/scArches-reproducibility> |  |
| CellxGene dataset | | Various cell types / organs | (141) |  |
| HUMAN-10k | | Human single cells | (119) | scInterpreter (119) |
| MOUSE-13k | | Mouse single cells |  |  |
| Nonenriched dataset | | Majority Rod photoreceptors (ROD) | https://doi.  org/10.5281/zenodo.14648190 | scGPT (120, 121) |
| AC-enriched dataset | | Amacrine cells (AC) |  |  |
| BC-enriched dataset | | Bipolar cells (BC) |  |  |
| RGC-enriched dataset | | Retinal ganglion cells (RGC) |  |  |
| Two age-related macular degeneration (AMD) datasets | | Mixed retinal cell types from human AMD samples |  |  |

**References**

1. Ma,F. and Pellegrini,M. (2020) ACTINN: Automated identification of cell types in single cell RNA sequencing. *Bioinformatics*, **36**, 533–538.

2. Cao,Z.J., Wei,L., Lu,S., Yang,D.C. and Gao,G. (2020) Searching large-scale scRNA-seq databases via unbiased cell embedding with Cell BLAST. *Nat Commun*, **11**, 3458.

3. Yin,Q. and Chen,L. (2024) CellTICS: An explainable neural network for cell-type identification and interpretation based on single-cell RNA-seq data. *Brief Bioinform*, **25**, bbad449.

4. Dong,S., Deng,K. and Huang,X. (2024) Single-cell type annotation with deep learning in 265 cell types for humans. *Bioinformatics Advances*, **4**, vbae054.

5. Goyal,M., Serrano,G., Argemi,J., Shomorony,I., Hernaez,M. and Ochoa,I. (2022) Gene expression JIND: joint integration and discrimination for automated single-cell annotation. *Bioinformatics*, **38**, 2488–2495.

6. Zhou,Z., Du,Z. and Chaterji,S. (2022) KRATOS: Context-Aware Cell Type Classification and Interpretation using Joint Dimensionality Reduction and Clustering. In *Proceedings of the ACM SIGKDD International Conference on Knowledge Discovery and Data Mining*. Association for Computing Machinery, pp. 2616–2625.

7. Johnson,T.S., Wang,T., Huang,Z., Yu,C.Y., Wu,Y., Han,Y., Zhang,Y., Huang,K. and Zhang,J. (2019) LAmbDA: Label ambiguous domain adaptation dataset integration reduces batch effects and improves subtype detection. *Bioinformatics*, **35**, 4696–4706.

8. Koh,W. and Hoon,S. (2021) MapCell: Learning a Comparative Cell Type Distance Metric With Siamese Neural Nets With Applications Toward Cell-Type Identification Across Experimental Datasets. *Front Cell Dev Biol*, **9**, 767897.

9. Ray,S. and Schönhuth,A. (2020) MarkerCapsule: Explainable Single Cell Typing using Capsule Networks. *bioRxiv preprint*, 10.1101/2020.09.22.307512.

10. Brbić,M., Zitnik,M., Wang,S., Pisco,A.O., Altman,R.B., Darmanis,S. and Leskovec,J. (2020) MARS: discovering novel cell types across heterogeneous single-cell experiments. *Nat Methods*, **17**, 1200–1206.

11. Liu,C., Huang,H. and Yang,P. (2023) Multi-task learning from multimodal single-cell omics with Matilda. *Nucleic Acids Res*, **51**, e45.

12. Xiong,Y.X., Wang,M.G., Chen,L. and Zhang,X.F. (2023) Cell-type annotation with accurate unseen cell-type identification using multiple references. *PLoS Comput Biol*, **19**, e1011261.

13. Wang,S., Pisco,A.O., McGeever,A., Brbic,M., Zitnik,M., Darmanis,S., Leskovec,J., Karkanias,J. and Altman,R.B. (2020) Unifying single-cell annotations based on the Cell Ontology. *bioRxiv preprint*, 10.1101/810234.

14. Yang,F., Wang,W., Wang,F., Fang,Y., Tang,D., Huang,J., Lu,H. and Yao,J. (2022) scBERT as a large-scale pretrained deep language model for cell type annotation of single-cell RNA-seq data. *Nat Mach Intell*, **4**, 852–866.

15. Wang,L., Nie,R., Yu,Z., Xin,R., Zheng,C., Zhang,Z., Zhang,J. and Cai,J. (2020) An interpretable deep-learning architecture of capsule networks for identifying cell-type gene expression programs from single-cell RNA-sequencing data. *Nat Mach Intell*, **2**, 693–703.

16. Shao,X., Yang,H., Zhuang,X., Liao,J., Yang,P., Cheng,J., Lu,X., Chen,H. and Fan,X. (2021) ScDeepSort: A pre-trained cell-type annotation method for single-cell transcriptomics using deep learning with a weighted graph neural network. *Nucleic Acids Res*, **49**, E122.

17. Kimmel,J.C. and Kelley,D.R. (2021) Semisupervised adversarial neural networks for single-cell classification. *Genome Res*, **31**, 1781–1793.

18. Zhang​,R., Luo​,Y., Ma​,J., Zhang​,M., Wang​,S. and Allen,P.G. (2022) scPretrain: Multi-task self-supervised learning for cell type classification. *Bioinformatics*, **38**, 1607–1614.

19. Yang,J., Wang,W. and Zhang,X. (2024) scSemiGCN: boosting cell-type annotation from noise-resistant graph neural networks with extremely limited supervision. *Bioinformatics*, **40**, btae091.

20. Fischer,F., Fischer,D.S., Mukhin,R., Isaev,A., Biederstedt,E., Villani,A.C. and Theis,F.J. (2024) scTab: Scaling cross-tissue single-cell annotation models. *Nat Commun*, **15**.

21. Ergen,C., Pour Amiri,V.V., Kim,M., Streets,A., Gayoso,A. and Yosef,N. (2024) Scvi-hub: an actionable repository for model-driven single cell analysis. *bioRxiv preprint*, 10.1101/2024.03.01.582887.

22. Gundogdu,P., Alamo,I., Nepomuceno-Chamorro,I.A., Dopazo,J. and Loucera,C. (2023) SigPrimedNet: A Signaling-Informed Neural Network for scRNA-seq Annotation of Known and Unknown Cell Types. *Biology (Basel)*, **12**, 579.

23. Xie,P., Gao,M., Wang,C., Zhang,J., Noel,P., Yang,C., Von Hoff,D., Han,H., Zhang,M.Q. and Lin,W. (2019) SuperCT: A supervised-learning framework for enhanced characterization of single-cell transcriptomic profiles. *Nucleic Acids Res*, **47**, e48.

24. Liu,Y., Wei,G., Li,C., Shen,L.C., Gasser,R.B., Song,J., Chen,D. and Yu,D.J. (2023) TripletCell: a deep metric learning framework for accurate annotation of cell types at the single-cell level. *Brief Bioinform*, **24**, bbad132.

25. Mädler,S.C., Julien-Laferriere,A., Wyss,L., Phan,M., Sonrel,A., Kang,A.S.W., Ulrich,E., Schmucki,R., Zhang,J.D., Ebeling,M., *et al.* (2021) Besca, a single-cell transcriptomics analysis toolkit to accelerate translational research. *NAR Genom Bioinform*, **3**, lqab102.

26. Newman,A.M., Liu,C.L., Green,M.R., Gentles,A.J., Feng,W., Xu,Y., Hoang,C.D., Diehn,M. and Alizadeh,A.A. (2015) Robust enumeration of cell subsets from tissue expression profiles. *Nat Methods*, **12**, 453–457.

27. Ran,R. and Brubaker,D.K. (2023) Enhanced annotation of CD45RA to distinguish T cell subsets in single-cell RNA-seq via machine learning. *Bioinformatics Advances*, **3**, vbad159.

28. Nguyen​,V., Griss​,J. and Griss,J. (2022) scClassifR: Framework to accurately classify cell types in single-cell RNA-sequencing data. *BMC Bioinformatics*, **23**, 44.

29. Michielsen,L., Reinders,M.J.T. and Mahfouz,A. (2021) Hierarchical progressive learning of cell identities in single-cell data. *Nat Commun*, **12**, 2799.

30. Alquicira-Hernandez,J., Sathe,A., Ji,H.P., Nguyen,Q. and Powell,J.E. (2019) ScPred: Accurate supervised method for cell-type classification from single-cell RNA-seq data. *Genome Biol*, **20**, 264.

31. Lieberman,Y., Rokach,L. and Shay,T. (2018) CaSTLe - Classification of single cells by transfer learning: Harnessing the power of publicly available single cell RNA sequencing experiments to annotate new experiments. *PLoS One*, **13**, e0205499.

32. Xu,C., Prete,M., Webb,S., Jardine,L., Stewart,B.J., Hoo,R., He,P., Meyer,K.B. and Teichmann,S.A. (2023) Automatic cell-type harmonization and integration across Human Cell Atlas datasets. *Cell*, **186**, 5876-5891.e20.

33. Cahan,P., Li,H., Morris,S.A., Lummertz Da Rocha,E., Daley,G.Q. and Collins,J.J. (2014) CellNet: Network biology applied to stem cell engineering. *Cell*, **158**, 903–915.

34. Wang,X., Chai,Z., Li,S., Liu,Y., Li,C., Jiang,Y. and Liu,Q. (2024) CTISL: a dynamic stacking multi-class classification approach for identifying cell types from single-cell RNA-seq data. *Bioinformatics*, **40**, btae063.

35. Kaymaz,Y., Ganglberger,F., Tang,M., Haslinger,C., Fernandez-Albert,F., Lawless,N. and Sackton,T.B. (2021) HieRFIT: A hierarchical cell type classification tool for projections from complex single-cell atlas datasets. *Bioinformatics*, **37**, 4431–4436.

36. Aybey,B., Zhao,S., Brors,B. and Staub,E. (2023) Immune cell type signature discovery and random forest classification for analysis of single cell gene expression datasets. *Front Immunol*, **14**, 1194745.

37. Xu,Y., Baumgart,S.J., Stegmann,C.M. and Hayat,S. (2022) MACA: Marker-based automatic cell-type annotation for single-cell expression data. *Bioinformatics*, **38**, 1756–1760.

38. Aevermann,B., Zhang,Y., Novotny,M., Keshk,M., Bakken,T., Miller,J., Hodge,R., Lelieveldt,B., Lein,E. and Scheuermann,R.H. (2021) A machine learning method for the discovery of minimum marker gene combinations for cell type identification from single-cell RNA sequencing. *Genome Res*, **31**, 1767–1780.

39. Ji,X., Tsao,D., Bai,K., Tsao,M., Xing,L. and Zhang,X. (2023) scAnnotate: an automated cell type annotation tool for single-cell RNA-sequencing data. *Bioinform Adv*, **3**, 30.

40. Lin,Y., Cao,Y., Kim,H.J., Salim,A., Speed,T.P., Lin,D.M., Yang,P. and Yang,J.Y.H. (2020) scClassify: sample size estimation and multiscale classification of cells using single and multiple reference. *Mol Syst Biol*, **16**, e9389.

41. Cao,Y., Lin,Y., Ormerod,J.T., Yang,P., Yang,J.Y.H. and Lo,K.K. (2019) ScDC: Single cell differential composition analysis. *BMC Bioinformatics*, **20**, 721.

42. Shen,Y., Chu,Q., Timko,M.P. and Fan,L. (2021) ScDetect: A rank-based ensemble learning algorithm for cell type identification of single-cell RNA sequencing in cancer. *Bioinformatics*, **37**, 4115–4122.

43. Yin,Q., Wang,Y., Guan,J. and Ji,G. (2022) scIAE: an integrative autoencoder-based ensemble classification framework for single-cell RNA-seq data. *Brief Bioinform*, **23**, bbab508.

44. Wan,S., Kim,J. and Won,K.J. (2020) SHARP: Single-cell RNA-seq Hyper-fast and Accurate Processing via Ensemble Random Projection. *Genome Res*, **30**, 205–213.

45. Tan,Y. and Cahan,P. (2019) SingleCellNet: A Computational Tool to Classify Single Cell RNA-Seq Data Across Platforms and Across Species. *Cell Syst*, **9**, 207-213.e2.

46. Shasha,C., Tian,Y., Mair,F., Miller,H.E.R. and Gottardo,R. (2021) Superscan: Supervised Single-Cell Annotation. *bioRxiv preprint*, 10.1101/2021.05.20.445014.

47. Srivastava,D., Iyer,A., Kumar,V. and Sengupta,D. (2018) CellAtlasSearch: A scalable search engine for single cells. *Nucleic Acids Res*, **46**, W141–W147.

48. Sato,K., Tsuyuzaki,K., Shimizu,K. and Nikaido,I. (2019) CellFishing.jl: An ultrafast and scalable cell search method for single-cell RNA sequencing. *Genome Biol*, **20**, 31.

49. Srinivasan,S., Leshchyk,A., Johnson,N.T. and Korkin,D. (2020) A hybrid deep clustering approach for robust cell type profiling using single-cell RNA-seq data. *RNA*, **26**, 1303–1319.

50. Zanini,F., Berghuis,B.A., Jones,R.C., Nicolis di Robilant,B., Nong,R.Y., Norton,J.A., Clarke,M.F. and Quake,S.R. (2020) Northstar enables automatic classification of known and novel cell types from tumor samples. *Sci Rep*, **10**, 15251.

51. Xu,C., Lopez,R., Mehlman,E., Regier,J., Jordan,M.I. and Yosef,N. (2021) Probabilistic harmonization and annotation of single‐cell transcriptomics data with deep generative models. *Mol Syst Biol*, **17**, 9620.

52. Zhang,Z., Luo,D., Zhong,X., Choi,J.H., Ma,Y., Wang,S., Mahrt,E., Guo,W., Stawiski,E.W., Modrusan,Z., *et al.* (2019) Scina: Semi-supervised analysis of single cells in silico. *Genes (Basel)*, **10**, 531.

53. Kiselev,V.Yu., Yiu,A. and Hemberg,M. (2018) scmap: projection of single-cell RNA-seq data across data sets. *Nat Methods*, **15**, 359–362.

54. Bernstein​,M.N., Ma​,Z., Gleicher​,M. and Dewey​,C.N. (2020) CellO: Comprehensive and hierarchical cell type classification of human cells with the Cell Ontology. *iScience* , **24**, 101913.

55. Domínguez C, Xu C, Jarvis LB, Rainbow DB, Wells SB, Gomes T, Howlett SK, Suchanek O, Polanski K, King HW, *et al.* (2022) Cross-tissue immune cell analysis reveals tissue-specific features in humans. *Science (1979)*, **376**, 5197.

56. Pliner,H.A., Shendure,J. and Trapnell,C. (2019) Supervised classification enables rapid annotation of cell atlases. *Nat Methods*, **16**, 983–986.

57. Torang,A., Gupta,P. and Klinke,D.J. (2019) An elastic-net logistic regression approach to generate classifiers and gene signatures for types of immune cells and T helper cell subsets. *BMC Bioinformatics*, **20**, 433.

58. Meng,G., Pan,Y., Tang,W., Zhang,L., Cui,Y., Schumacher,F.R., Wang,M., Wang,R., He,S., Krischer,J., *et al.* (2024) imply: improving cell-type deconvolution accuracy using personalized reference profiles. *Genome Med*, **16**, 559579.

59. Heiling,H.M., Wilson,D.R., Rashid,N.U., Sun,W. and Ibrahim,J.G. (2023) Estimating cell type composition using isoform expression one gene at a time. *Biometrics*, **79**, 854–865.

60. Li,C., Liu,B., Kang,B., Liu,Z., Liu,Y., Chen,C., Ren,X. and Zhang,Z. (2020) SciBet as a portable and fast single cell type identifier. *Nat Commun*, **11**, 1818.

61. Li,D., Ding,J. and Bar-Joseph,Z. (2022) UNIFAN: A Tool for Unsupervised Single-Cell Clustering and Annotation. *Journal of Computational Biology*, **29**, 1229–1232.

62. Kim H, Lee J, Kang K and Yoon S. (2022) MarkerCount: A stable, count-based cell type identifier for single-cell RNA-seq experiments. *Comput Struct Biotechnol*, **20**, 3120–3132.

63. Zhai,Y., Chen,L. and Deng,M. (2024) ScEVOLVE: cell-type incremental annotation without forgetting for single-cell RNA-seq data. *Brief Bioinform*, **25**, bbae039.

64. Choi,J.-H., In Kim,H. and Woo,H.G. (2020) scTyper: a comprehensive pipeline for the cell typing analysis of single-cell RNA-seq data. *BMC Bioinformatics*, **21**, 342.

65. Zhang,A.W., O’Flanagan,C., Chavez,E.A., Lim,J.L.P., Ceglia,N., McPherson,A., Wiens,M., Walters,P., Chan,T., Hewitson,B., *et al.* (2019) Probabilistic cell-type assignment of single-cell RNA-seq for tumor microenvironment profiling. *Nat Methods*, **16**, 1007–1015.

66. de Kanter,J.K., Lijnzaad,P., Candelli,T., Margaritis,T. and Holstege,F.C.P. (2019) CHETAH: a selective, hierarchical cell type identification method for single-cell RNA sequencing. *Nucleic Acids Res*, **47**, E95.

67. Wagner,F. and Yanai,I. (2018) Moana: A robust and scalable cell type classification framework for single-cell RNA-Seq data. *bioRxiv preprint*, 10.1101/456129.

68. Grün,D., Muraro,M.J., Boisset,J.C., Wiebrands,K., Lyubimova,A., Dharmadhikari,G., van den Born,M., van Es,J., Jansen,E., Clevers,H., *et al.* (2016) De Novo Prediction of Stem Cell Identity using Single-Cell Transcriptome Data. *Cell Stem Cell*, **19**, 266–277.

69. Fu,R., Gillen,A.E., Sheridan,R.M., Tian,C., Daya,M., Hao,Y., Hesselberth,J.R. and Riemondy,K.A. (2020) clustifyr: an R package for automated single-cell RNA sequencing cluster classification. *F1000Res*, **9**, 223.

70. Pei,G., Yan,F., Simon,L.M., Dai,Y., Jia,P. and Zhao,Z. (2023) deCS: A Tool for Systematic Cell Type Annotations of Single-cell RNA Sequencing Data among Human Tissues. *Genomics Proteomics Bioinformatics*, **21**, 370–384.

71. Shao X, Liao J, Lu X, Xue R, Ai N and Fan X. (2020) scCATCH: Automatic Annotation on Cell Types of Clusters from Single-Cell RNA Sequencing Data. *iScience*, **23**, 100882.

72. Boufea,K., Seth,S. and Batada,N.N. (2020) scID Uses Discriminant Analysis to Identify Transcriptionally Equivalent Cell Types across Single-Cell RNA-Seq Data with Batch Effect. *iScience*, **23**, 100914.

73. Hou,R., Denisenko,E. and Forrest,A.R.R. (2019) ScMatch: A single-cell gene expression profile annotation tool using reference datasets. *Bioinformatics*, **35**, 4688–4695.

74. Yang,L., Ng,Y.E., Sun,H., Li,Y., Chini,L.C.S., LeBrasseur,N.K., Chen,J. and Zhang,X. (2023) Single-cell Mayo Map (scMayoMap): an easy-to-use tool for cell type annotation in single-cell RNA-sequencing data analysis. *BMC Biol*, **21**, 223.

75. Sun,H., Zhou,Y., Fei,L., Chen,H. and Guo,G. (2019) scMCA: A tool to define mouse cell types based on single-cell digital expression. In *Methods in Molecular Biology*. Humana Press Inc., Vol. 1935, pp. 91–96.

76. Prummer,M., Bertolini,A., Bosshard,L., Barkmann,F., Yates,J., Boeva,V., Tumor Profiler Consortium,T., Stekhoven,D. and Singer,F. (2023) scROSHI: robust supervised hierar chical identification of single cells. *NAR Genom Bioinform*, **5**, lqad058.

77. Cao,Y., Wang,X. and Peng,G. (2020) SCSA: A cell type annotation tool for single-cell RNA-seq data. *Front Genet*, **11**, 490.

78. Aran,D., Looney,A.P., Liu,L., Wu,E., Fong,V., Hsu,A., Chak,S., Naikawadi,R.P., Wolters,P.J., Abate,A.R., *et al.* (2019) Reference-based analysis of lung single-cell sequencing reveals a transitional profibrotic macrophage. *Nat Immunol*, **20**, 163–172.

79. Xu,J., Zhang,A., Liu,F., Chen,L. and Zhang,X. (2023) CIForm as a Transformer-based model for cell-type annotation of large-scale single-cell RNA-seq data. *Brief Bioinform*, **24**, bbad195.

80. Hu,J., Li,X., Hu,G., Lyu,Y., Susztak,K. and Li,M. (2020) Iterative transfer learning with neural network for clustering and cell type classification in single-cell RNA-seq analysis. *Nat Mach Intell*, **2**, 607–618.

81. Upadhyay,P. and Ray,S. (2022) A Regularized Multi-Task Learning Approach for Cell Type Detection in Single-Cell RNA Sequencing Data. *Front Genet*, **13**, 788832.

82. Bravo González-Blas,C., De Winter,S., Hulselmans,G., Hecker,N., Matetovici,I., Christiaens,V., Poovathingal,S., Wouters,J., Aibar,S. and Aerts,S. (2023) SCENIC+: single-cell multiomic inference of enhancers and gene regulatory networks. *Nat Methods*, **20**, 1355–1367.

83. Zhai,Y., Chen,L. and Deng,M. (2023) scGAD: a new task and end-to-end framework for generalized cell type annotation and discovery. *Brief Bioinform*, **24**, bbad045.

84. Duan,B., Zhu,C., Chuai,G., Tang,C., Chen,X., Chen,S., Fu,S., Li,G. and Liu,Q. (2020) Learning for single-cell assignment. *Sci. Adv*, **6**, eabd0855.

85. Jiao,L., Wang,G., Dai,H., Li,X., Wang,S. and Song,T. (2023) scTransSort: Transformers for Intelligent Annotation of Cell Types by Gene Embeddings. *Biomolecules*, **13**, 611.

86. Satija,R., Farrell,J.A., Gennert,D., Schier,A.F. and Regev,A. (2015) Spatial reconstruction of single-cell gene expression data. *Nat Biotechnol*, **33**, 495–502.

87. Chen,J., Xu,H., Tao,W., Chen,Z., Zhao,Y. and Han,J.D.J. (2023) Transformer for one stop interpretable cell type annotation. *Nat Commun*, **14**, 223.

88. Song,T., Dai,H., Wang,S., Wang,G., Zhang,X., Zhang,Y. and Jiao,L. (2022) TransCluster: A Cell-Type Identification Method for single-cell RNA-Seq data using deep learning based on transformer. *Front Genet*, **13**, 1038919.

89. Domanskyi,S., Hakansson,A., Bertus,T.J., Paternostro,G. and Piermarocchi,C. (2019) Digital Cell Sorter (DCS): a cell type identification, anomaly detection, and Hopfield landscapes toolkit for single-cell transcriptomics. *BMC Bioinformatics*, **9**, 10670.

90. Liu,X., Gosline,S.J.C., Pflieger,L.T., Wallet,P., Iyer,A., Guinney,J., Bild,A.H. and Chang,J.T. (2021) Knowledge-based classification of fine-grained immune cell types in single-cell RNA-Seq data. *Brief Bioinform*, **22**, bbab039.

91. Liu,B., Wu,F.X. and Zou,X. (2021) ScASK: A Novel Ensemble Framework for Classifying Cell Types Based on Single-cell RNA-seq Data. *IEEE J Biomed Health Inform*, **25**, 3230–3239.

92. Kim,T., Lo,K., Geddes,T.A., Kim,H.J., Yang,J.Y.H. and Yang,P. (2019) ScReClassify: Post hoc cell type classification of single-cell RNA-seq data. *BMC Genomics*, **20**, 913.

93. Nouri,N., Gaglia,G., Kurlovs,A.H., de Rinaldis,E. and Savova,V. (2023) A marker gene-based method for identifying the cell-type of origin from single-cell RNA sequencing data. *MethodsX*, **10**, 102196.

94. Ianevski,A., Giri,A.K. and Aittokallio,T. (2022) Fully-automated and ultra-fast cell-type identification using specific marker combinations from single-cell transcriptomic data. *Nat Commun*, **13**, 1246.

95. Kim,N., Kim,H.K., Lee,K., Hong,Y., Cho,J.H., Choi,J.W., Lee,J.-I., Suh,Y.-L., Ku,B.M., Eum,H.H., *et al.* (2020) Single-cell RNA sequencing demonstrates the molecular and cellular reprogramming of metastatic lung adenocarcinoma. *Nat Commun*, **11**, 2285.

96. Zheng,G.X.Y., Terry,J.M., Belgrader,P., Ryvkin,P., Bent,Z.W., Wilson,R., Ziraldo,S.B., Wheeler,T.D., McDermott,G.P., Zhu,J., *et al.* (2017) Massively parallel digital transcriptional profiling of single cells. *Nat Commun*, **8**, 14049.

97. Baron M, Veres A, Wolock SL, Faust AL, Gaujoux R, Vetere A, Ryu JH, Wagner BK, Shen-Orr SS, Klein AM, *et al.* (2016) A Single-Cell Transcriptomic Map of the Human and Mouse Pancreas Reveals Inter- and Intra-cell Population Structure. *Cell Syst*, **3**, 346–360.

98. Muraro,M.J., Dharmadhikari,G., Grün,D., Groen,N., Dielen,T., Jansen,E., van Gurp,L., Engelse,M.A., Carlotti,F., de Koning,E.J.P., *et al.* (2016) A Single-Cell Transcriptome Atlas of the Human Pancreas. *Cell Syst*, **3**, 385-394.e3.

99. Xin,Y., Kim,J., Okamoto,H., Ni,M., Wei,Y., Adler,C., Murphy,A.J., Yancopoulos,G.D., Lin,C. and Gromada,J. (2016) RNA Sequencing of Single Human Islet Cells Reveals Type 2 Diabetes Genes. *Cell Metab*, **24**, 608–615.

100. Segerstolpe,Å., Palasantza,A., Eliasson,P., Andersson,E.M., Andréasson,A.C., Sun,X., Picelli,S., Sabirsh,A., Clausen,M., Bjursell,M.K., *et al.* (2016) Single-Cell Transcriptome Profiling of Human Pancreatic Islets in Health and Type 2 Diabetes. *Cell Metab*, **24**, 593–607.

101. Tian,L., Dong,X., Freytag,S., Lê Cao,K.-A., Su,S., JalalAbadi,A., Amann-Zalcenstein,D., Weber,T.S., Seidi,A., Jabbari,J.S., *et al.* (2019) Benchmarking single cell RNA-sequencing analysis pipelines using mixture control experiments. *Nat Methods*, **16**, 479–487.

102. Lawlor,N., George,J., Bolisetty,M., Kursawe,R., Sun,L., Sivakamasundari,V., Kycia,I., Robson,P. and Stitzel,M.L. (2017) Single-cell transcriptomes identify human islet cell signatures and reveal cell-type-specific expression changes in type 2 diabetes. *Genome Res*, **27**, 208–222.

103. Anderson,A.G., Kulkarni,A., Harper,M. and Konopka,G. (2020) Single-Cell Analysis of Foxp1-Driven Mechanisms Essential for Striatal Development. *Cell Rep*, **30**, 3051-3066.e7.

104. Jessa,S., Blanchet-Cohen,A., Krug,B., Vladoiu,M., Coutelier,M., Faury,D., Poreau,B., De Jay,N., Hébert,S., Monlong,J., *et al.* (2019) Stalled developmental programs at the root of pediatric brain tumors. *Nat Genet*, **51**, 1702–1713.

105. Vladoiu,M.C., El-Hamamy,I., Donovan,L.K., Farooq,H., Holgado,B.L., Sundaravadanam,Y., Ramaswamy,V., Hendrikse,L.D., Kumar,S., Mack,S.C., *et al.* (2019) Childhood cerebellar tumours mirror conserved fetal transcriptional programs. *Nature*, **572**, 67–73.

106. Schaum,N., Karkanias,J., Neff,N.F., May,A.P., Quake,S.R., Wyss-Coray,T., Darmanis,S., Batson,J., Botvinnik,O., Chen,M.B., *et al.* (2018) Single-cell transcriptomics of 20 mouse organs creates a Tabula Muris. *Nature*, **562**, 367–372.

107. Selewa,A., Dohn,R., Eckart,H., Lozano,S., Xie,B., Gauchat,E., Elorbany,R., Rhodes,K., Burnett,J., Gilad,Y., *et al.* (2019) Systematic Comparison of High-throughput Single-Cell and Single-Nucleus Transcriptomes during Cardiomyocyte Differentiation. *bioRxiv preprint*, 10.1101/585901.

108. Zhao,X., Wu,S., Fang,N., Sun,X. and Fan,J. (2020) Evaluation of single-cell classifiers for single-cell RNA sequencing data sets. *Brief Bioinform*, **21**, 1581–1595.

109. Huang,Q., Liu,Y., Du,Y. and Garmire,L.X. (2021) Evaluation of Cell Type Annotation R Packages on Single-cell RNA-seq Data. *Genomics Proteomics Bioinformatics*, **19**, 267–281.

110. Ye,W., Xiang,J., Ma,Y., Liang,H., Wang,T., Xiang,Q., Peng Xiang,A., Li,W. and Huang,W. (2024) Objectively Evaluating the Reliability of Cell Type Annotation Using LLM-Based Strategies. *arXiv preprint*, 10.48550/arXiv.2409.15678.

111. Hou,W. and Ji,Z. (2024) Assessing GPT-4 for cell type annotation in single-cell RNA-seq analysis. *Nat Methods*, **21**, 1462–1465.

112. Xiao,Y., Liu,J., Zheng,Y., Xie,X., Hao,J., Li,M., Wang,R., Ni,F., Li,Y., Luo,J., *et al.* (2024) CellAgent: An LLM-driven Multi-Agent Framework for Automated Single-cell Data Analysis. *arXiv preprint*, 10.48550/arXiv.2407.09811.

113. Xie,E., Cheng,L., Shireman,J., Cai,Y., Liu,J., Mohanty,C., Dey,M. and Kendziorski,C. (2024) CASSIA: a multi-agent large language model for reference free, interpretable, and automated cell annotation of single-cell RNA-sequencing data. *bioRxiv preprint*, 10.1101/2024.12.04.626476.

114. Zhao,H., Liu,Y., Yao,J., Xiong,L., Zhou,Z. and Zhang,Z. (2025) Celler:A Genomic Language Model for Long-Tailed Single-Cell Annotation. *arXiv preprint*, 10.48550/arXiv.2504.00020.

115. Cao,G., Shen,Y., Wu,J., Chao,H., Chen,M. and Chen,D. (2025) CellReasoner: A reasoning-enhanced large language model for cell type annotation. *bioRxiv preprint*, 10.1101/2025.05.20.655112.

116. Chen,J., Zhang,J., Yao,H. and Li,Y. (2025) CellTypeAgent: Trustworthy cell type annotation with Large Language Models. *arXiv preprint*, 10.48550/arXiv.2505.08844.

117. Han,D., Jia,Y., Chen,R., Han,W., Guo,S. and Wang,J. (2025) ReCellTy: Domain-specific knowledge graph retrieval-augmented LLMs workflow for single-cell annotation. *arXiv preprint*, 10.48550/arXiv.2505.00017.

118. Wu,Y. and Tang,F. (2025) scExtract: leveraging large language models for fully automated single-cell RNA-seq data annotation and prior-informed multi-dataset integration. *Genome Biol*, **26**, 174.

119. Li,C., Xiao,M., Wang,P., Feng,G., Li,X. and Zhou,Y. (2024) scInterpreter: Training Large Language Models to Interpret scRNA-seq Data for Cell Type Annotation. *arXiv preprint*, 10.48550/arXiv.2402.12405.

120. Ding,S., Li,J., Luo,R., Cui,H., Wang,B. and Chen,R. (2025) scGPT: end-to-end protocol for fine-tuned retinal cell type annotation. *Nat Protoc*, 10.1038/s41596-025-01220-1.

121. Cui,H., Wang,C., Maan,H., Pang,K., Luo,F., Duan,N. and Wang,B. (2024) scGPT: toward building a foundation model for single-cell multi-omics using generative AI. *Nat Methods*, **21**, 1470–1480.

122. Hao,Y., Hao,S., Andersen-Nissen,E., Mauck,W.M., Zheng,S., Butler,A., Lee,M.J., Wilk,A.J., Darby,C., Zagar,M., *et al.* (2020) Integrated analysis of multimodal single-cell data. *Cell*, **184, no. 13**, 3573-3587.e29.

123. Buechler,M.B., Pradhan,R.N., Krishnamurty,A.T., Cox,C., Calviello,A.K., Wang,A.W., Yang,Y.A., Tam,L., Caothien,R., Roose-Girma,M., *et al.* (2021) Cross-tissue organization of the fibroblast lineage. *Nature*, **593**, 575–579.

124. Kang,B., Camps,J., Fan,B., Jiang,H., Ibrahim,M.M., Hu,X., Qin,S., Kirchhoff,D., Chiang,D.Y., Wang,S., *et al.* (2022) Parallel single-cell and bulk transcriptome analyses reveal key features of the gastric tumor microenvironment. *Genome Biol*, **23**, 265.

125. Tyser,R.C.V., Mahammadov,E., Nakanoh,S., Vallier,L., Scialdone,A. and Srinivas,S. (2021) Single-cell transcriptomic characterization of a gastrulating human embryo. *Nature*, **600**, 285–289.

126. Han,X., Wang,R., Zhou,Y., Fei,L., Sun,H., Lai,S., Saadatpour,A., Zhou,Z., Chen,H., Ye,F., *et al.* (2018) Mapping the Mouse Cell Atlas by Microwell-Seq. *Cell*, **172**, 1091-1107.e17.

127. Han,X., Zhou,Z., Fei,L., Sun,H., Wang,R., Chen,Y., Chen,H., Wang,J., Tang,H., Ge,W., *et al.* (2020) Construction of a human cell landscape at single-cell level. *Nature*, **581**, 303–309.

128. Kim,N., Kim,H.K., Lee,K., Hong,Y., Cho,J.H., Choi,J.W., Lee,J. Il, Suh,Y.L., Ku,B.M., Eum,H.H., *et al.* (2020) Single-cell RNA sequencing demonstrates the molecular and cellular reprogramming of metastatic lung adenocarcinoma. *Nat Commun*, **11**, 2285.

129. Lee,H.O., Hong,Y., Etlioglu,H.E., Cho,Y.B., Pomella,V., Van den Bosch,B., Vanhecke,J., Verbandt,S., Hong,H., Min,J.W., *et al.* (2020) Lineage-dependent gene expression programs influence the immune landscape of colorectal cancer. *Nat Genet*, **52**, 594–603.

130. Chen,D., Sun,J., Zhu,J., Ding,X., Lan,T., Wang,X., Wu,W., Ou,Z., Zhu,L., Ding,P., *et al.* (2021) Single cell atlas for 11 non-model mammals, reptiles and birds. *Nat Commun*, **12**, 7083.

131. Eraslan,G., Drokhlyansky,E., Anand,S., Fiskin,E., Subramanian,A., Slyper,M., Wang,J., Wittenberghe,N. Van, Rouhana,J.M., Waldman,J., *et al.* (2022) Single-nucleus cross-tissue molecular reference maps toward understanding disease gene function. *Science (1979)*, **376**.

132. Tabula Sapiens Consortium*, Jones,R.C., Karkanias,J., Krasnow,M.A., Pisco,A.O., Quake,S.R., Salzman,J., Yosef,N., Bulthaup,B., Brown,P., *et al.* (2022) The Tabula Sapiens: A multiple-organ, single-cell transcriptomic atlas of humans. *Science*, **376**, eabl4896.

133. Liu,N., Jiang,C., Yao,X., Fang,M., Qiao,X., Zhu,L., Yang,Z., Gao,X., Ji,Y., Niu,C., *et al.* (2023) Single-cell landscape of primary central nervous system diffuse large B-cell lymphoma. *Cell Discov*, **9**, 55.

134. Schirmer,L., Velmeshev,D., Holmqvist,S., Kaufmann,M., Werneburg,S., Jung,D., Vistnes,S., Stockley,J.H., Young,A., Steindel,M., *et al.* (2019) Neuronal vulnerability and multilineage diversity in multiple sclerosis. *Nature*, **573**, 75–82.

135. MacParland,S.A., Liu,J.C., Ma,X.Z., Innes,B.T., Bartczak,A.M., Gage,B.K., Manuel,J., Khuu,N., Echeverri,J., Linares,I., *et al.* (2018) Single cell RNA sequencing of human liver reveals distinct intrahepatic macrophage populations. *Nat Commun*, **9**, 4383.

136. Litviňuková,M., Talavera-López,C., Maatz,H., Reichart,D., Worth,C.L., Lindberg,E.L., Kanda,M., Polanski,K., Heinig,M., Lee,M., *et al.* (2020) Cells of the adult human heart. *Nature*, **588**, 466–472.

137. Tucker,N.R., Chaffin,M., Fleming,S.J., Hall,A.W., Parsons,V.A., Bedi,K.C., Akkad,A.D., Herndon,C.N., Arduini,A., Papangeli,I., *et al.* (2020) Transcriptional and Cellular Diversity of the Human Heart. *Circulation*, **142**, 466–482.

138. Lukassen,S., Chua,R.L., Trefzer,T., Kahn,N.C., Schneider,M.A., Muley,T., Winter,H., Meister,M., Veith,C., Boots,A.W., *et al.* (2020) SARS-CoV-2 receptor ACE2 and TMPRSS2 are primarily expressed in bronchial transient secretory cells. *EMBO J*, **39**, e105114.

139. He,S., Wang,L.H., Liu,Y., Li,Y.Q., Chen,H.T., Xu,J.H., Peng,W., Lin,G.W., Wei,P.P., Li,B., *et al.* (2020) Single-cell transcriptome profiling of an adult human cell atlas of 15 major organs. *Genome Biol*, **21**, 294.

140. Reynolds,G., Vegh,P., Fletcher,J., Poyner,E.F.M., Stephenson,E., Goh,I., Botting,R.A., Huang,N., Olabi,B., Dubois,A., *et al.* (2021) Developmental cell programs are co-opted in inflammatory skin disease. *Science*, **371**.

141. Megill,C., Martin,B., Weaver,C., Bell,S., Prins,L., Badajoz,S., McCandless,B., Pisco,A.O., Kinsella,M., Griffin,F., *et al.* (2021) cellxgene: a performant, scalable exploration platform for high dimensional sparse matrices. 10.1101/2021.04.05.438318.
